# Supplementary material for: Individual canopy tree species maps for the National Ecological Observatory Network
Source: PLoS Biol. 2024 Jul 16;22(7):e3002700. doi: 10.1371/journal.pbio.3002700 (PMC11251727; doi:10.1371/journal.pbio.3002700)
Supplement: S1 File — Table A. Species included in each model for each NEON site. The number of samples (n) for each species in the canopy filtered data. To be included in the model, a species needs to have at least 10 training samples and 10 test samples at a site in the final filtered data. The number of predicted trees at each site, the proportion of total predictions at the site, and the rank abundance of each species is shown. Fig A. An example model architecture for data fusion between 1 m HSI data and 10 cm RGB for tree species classification. In this example, a batch of crowns (n = 20), each with an HSI and RGB pair, is run through the network to jointly predict tree classes (n = 10). The RGB model was a resnet-50 pretrained backbone, a common RGB architecture for image-classification. The HSI architecture was the same spectral attention network used throughout the rest of the paper. The 2 features were min-max normalized separately before combined and a joint classifier was used to predict tree species classes. Table B. Experiments comparing RGB, HSI, and joint model for a single NEON site (OSBS). The experiments were done without the hierarchical model or multi-temporal ensemble approaches to highlight the difference solely from source data type. Fig B. Comparison of site-level performance for modeling workflows that use training data solely from a single site (“per-site”) and pool training data across all sites “NEON-wide.” Micro averaged recall is the proportion of correctly predicted ground truth stems. Macro-averaged recall is the average recall per species, thereby weighing all species equally regardless of abundance. Several sites (JERC, MOAB, SCBI) lacked site-level predictions because the sample size per species at the individual site was too low. For the underlying data, see S4 Data. Fig C. Predicted canopy trees versus the count of all field measured trees in the NEON Woody Vegetation Structure plots. For each NEON site, the number of tree detections in the prediction [file pbio.3002700.s006.docx]

Table A: Species included in each model for each NEON site. The number of samples (n) for each species in the canopy filtered data. To be included in the model, a species needs to have at least 10 training samples and 10 test samples at a site in the final filtered data. The number of predicted trees at each site, the proportion of total predictions at the site, and the rank abundance of each species is shown.

| sci_name | count | site | proportion | rank | Habitat |
| --- | --- | --- | --- | --- | --- |
| Tsuga canadensis (L.) Carrire | 1916656 | BART | 0.44 | 1 | Northern Broadleaf |
| Fagus grandifolia Ehrh. | 557790 | BART | 0.13 | 2 | Northern Broadleaf |
| Acer saccharum Marshall | 529581 | BART | 0.12 | 3 | Northern Broadleaf |
| Betula alleghaniensis Britton | 527648 | BART | 0.12 | 4 | Northern Broadleaf |
| Acer rubrum L. | 384249 | BART | 0.09 | 5 | Northern Broadleaf |
| Fraxinus americana L. | 317331 | BART | 0.07 | 6 | Northern Broadleaf |
| Betula papyrifera Marshall | 119675 | BART | 0.03 | 7 | Northern Broadleaf |
| Juglans nigra L. | 856379 | BLAN | 0.35 | 1 | Southern Broadleaf |
| Cornus florida L. | 435672 | BLAN | 0.18 | 2 | Southern Broadleaf |
| Celtis occidentalis L. | 348581 | BLAN | 0.14 | 3 | Southern Broadleaf |
| Quercus rubra L. | 260101 | BLAN | 0.11 | 4 | Southern Broadleaf |
| Liriodendron tulipifera L. | 217721 | BLAN | 0.09 | 5 | Southern Broadleaf |
| Pinus strobus L. | 202535 | BLAN | 0.08 | 6 | Southern Broadleaf |
| Platanus occidentalis L. | 92087 | BLAN | 0.04 | 7 | Southern Broadleaf |
| Picea mariana (Mill.) Britton, Sterns & Poggenb. | 3858957 | BONA | 0.51 | 1 | Conifer |
| Betula neoalaskana Sarg. | 2365676 | BONA | 0.31 | 2 | Conifer |
| Populus tremuloides Michx. | 923889 | BONA | 0.12 | 3 | Conifer |
| Picea glauca (Moench) Voss | 424178 | BONA | 0.06 | 4 | Conifer |
| Quercus stellata Wangenh. | 1276070 | CLBJ | 0.60 | 1 | Savannah |
| Juniperus virginiana L. | 488138 | CLBJ | 0.23 | 2 | Savannah |
| Quercus marilandica M√ºnchh. | 375595 | CLBJ | 0.18 | 3 | Savannah |
| Picea glauca (Moench) Voss | 1282877 | DEJU | 0.37 | 1 | Conifer |
| Picea mariana (Mill.) Britton, Sterns & Poggenb. | 1223419 | DEJU | 0.36 | 2 | Conifer |
| Populus tremuloides Michx. | 920182 | DEJU | 0.27 | 3 | Conifer |
| Pinus taeda L. | 786987 | DELA | 0.22 | 1 | Southern Broadleaf |
| Quercus nigra L. | 756838 | DELA | 0.21 | 2 | Southern Broadleaf |
| Acer rubrum L. | 675465 | DELA | 0.19 | 3 | Southern Broadleaf |
| Liquidambar styraciflua L. | 478992 | DELA | 0.13 | 4 | Southern Broadleaf |
| Fraxinus pennsylvanica Marshall | 370191 | DELA | 0.10 | 5 | Southern Broadleaf |
| Celtis laevigata Willd. | 289826 | DELA | 0.08 | 6 | Southern Broadleaf |
| Carya tomentosa (Lam.) Nutt. | 262398 | DELA | 0.07 | 7 | Southern Broadleaf |
| Acer rubrum L. | 1142770 | GRSM | 0.47 | 1 | Southern Broadleaf |
| Quercus montana Willd. | 851493 | GRSM | 0.35 | 2 | Southern Broadleaf |
| Liriodendron tulipifera L. | 453621 | GRSM | 0.19 | 3 | Southern Broadleaf |
| Acer rubrum L. | 2092750 | HARV | 0.23 | 1 | Northern Broadleaf |
| Pinus strobus L. | 1928207 | HARV | 0.21 | 2 | Northern Broadleaf |
| Quercus rubra L. | 1656996 | HARV | 0.18 | 3 | Northern Broadleaf |
| Quercus alba L. | 1192530 | HARV | 0.13 | 4 | Northern Broadleaf |
| Betula lenta L. | 743261 | HARV | 0.08 | 5 | Northern Broadleaf |
| Fraxinus americana L. | 561297 | HARV | 0.06 | 6 | Northern Broadleaf |
| Tsuga canadensis (L.) Carri√re | 419849 | HARV | 0.05 | 7 | Northern Broadleaf |
| Betula alleghaniensis Britton | 257040 | HARV | 0.03 | 8 | Northern Broadleaf |
| Pinus resinosa Aiton | 91640 | HARV | 0.01 | 9 | Northern Broadleaf |
| Fagus grandifolia Ehrh. | 68370 | HARV | 0.01 | 10 | Northern Broadleaf |
| Prunus serotina Ehrh. | 55833 | HARV | 0.01 | 11 | Northern Broadleaf |
| Picea abies (L.) Karst. | 27501 | HARV | 0.00 | 12 | Northern Broadleaf |
| Nyssa sylvatica Marshall | 26159 | HARV | 0.00 | 13 | Northern Broadleaf |
| Quercus velutina Lam. | 1259 | HARV | 0.00 | 14 | Northern Broadleaf |
| Pinus palustris Mill. | 2159896 | JERC | 0.58 | 1 | Savannah |
| Quercus hemisphaerica W. Bartram ex Willd. | 1280439 | JERC | 0.35 | 2 | Savannah |
| Quercus margaretta | 257687 | JERC | 0.07 | 3 | Savannah |
| Liquidambar styraciflua L. | 2153931 | LENO | 0.63 | 1 | Southern Broadleaf |
| Quercus pagoda Raf. | 886055 | LENO | 0.26 | 2 | Southern Broadleaf |
| Quercus nigra L. | 396110 | LENO | 0.12 | 3 | Southern Broadleaf |
| Quercus rubra L. | 1610301 | MLBS | 0.43 | 1 | Southern Broadleaf |
| Acer rubrum L. | 873498 | MLBS | 0.23 | 2 | Southern Broadleaf |
| Quercus alba L. | 475352 | MLBS | 0.13 | 3 | Southern Broadleaf |
| Liriodendron tulipifera L. | 411167 | MLBS | 0.11 | 4 | Southern Broadleaf |
| Quercus coccinea M√ºnchh. | 359546 | MLBS | 0.10 | 5 | Southern Broadleaf |
| Pinus contorta Douglas ex Loudon | 2005920 | NIWO | 0.43 | 1 | Conifer |
| Picea engelmannii Parry ex Engelm. | 1268215 | NIWO | 0.27 | 2 | Conifer |
| Abies lasiocarpa (Hook.) Nutt. | 999052 | NIWO | 0.22 | 3 | Conifer |
| Pinus flexilis James | 354697 | NIWO | 0.08 | 4 | Conifer |
| Quercus hemisphaerica W. Bartram ex Willd. | 464452 | OSBS | 0.13 | 1 | Savannah |
| Pinus palustris Mill. | 459380 | OSBS | 0.13 | 2 | Savannah |
| Pinus taeda L. | 441837 | OSBS | 0.13 | 3 | Savannah |
| Pinus elliottii Engelm. | 428306 | OSBS | 0.12 | 4 | Savannah |
| Quercus laevis Walter | 315498 | OSBS | 0.09 | 5 | Savannah |
| Quercus geminata Small | 274916 | OSBS | 0.08 | 6 | Savannah |
| Quercus virginiana Mill. | 228768 | OSBS | 0.07 | 7 | Savannah |
| Pinus clausa (Chapm. ex Engelm.) Vasey ex Sarg. | 189431 | OSBS | 0.05 | 8 | Savannah |
| Liquidambar styraciflua L. | 162751 | OSBS | 0.05 | 9 | Savannah |
| Nyssa sylvatica Marshall | 145971 | OSBS | 0.04 | 10 | Savannah |
| Magnolia sp. | 132960 | OSBS | 0.04 | 11 | Savannah |
| Quercus nigra L. | 84128 | OSBS | 0.02 | 12 | Savannah |
| Acer rubrum L. | 73579 | OSBS | 0.02 | 13 | Savannah |
| Carya glabra (Mill.) Sweet | 61204 | OSBS | 0.02 | 14 | Savannah |
| Pinus contorta Douglas ex Loudon | 2656483 | RMNP | 0.35 | 1 | Conifer |
| Pinus ponderosa Lawson & C. Lawson | 1491041 | RMNP | 0.20 | 2 | Conifer |
| Pseudotsuga menziesii (Mirb.) Franco | 1185424 | RMNP | 0.16 | 3 | Conifer |
| Pinus flexilis James | 812500 | RMNP | 0.11 | 4 | Conifer |
| Populus tremuloides Michx. | 513952 | RMNP | 0.07 | 5 | Conifer |
| Picea engelmannii Parry ex Engelm. | 419298 | RMNP | 0.06 | 6 | Conifer |
| Abies lasiocarpa (Hook.) Nutt. | 408857 | RMNP | 0.05 | 7 | Conifer |
| Liriodendron tulipifera L. | 614569 | SERC | 0.27 | 1 | Southern Broadleaf |
| Liquidambar styraciflua L. | 503363 | SERC | 0.22 | 2 | Southern Broadleaf |
| Acer rubrum L. | 256490 | SERC | 0.11 | 3 | Southern Broadleaf |
| Quercus falcata Michx. | 236338 | SERC | 0.10 | 4 | Southern Broadleaf |
| Fraxinus pennsylvanica Marshall | 179711 | SERC | 0.08 | 5 | Southern Broadleaf |
| Platanus occidentalis L. | 130393 | SERC | 0.06 | 6 | Southern Broadleaf |
| Quercus velutina Lam. | 117590 | SERC | 0.05 | 7 | Southern Broadleaf |
| Quercus alba L. | 91438 | SERC | 0.04 | 8 | Southern Broadleaf |
| Fagus grandifolia Ehrh. | 87899 | SERC | 0.04 | 9 | Southern Broadleaf |
| Carya tomentosa (Lam.) Nutt. | 44351 | SERC | 0.02 | 10 | Southern Broadleaf |
| Quercus wislizeni A. DC. | 410332 | SJER | 0.48 | 1 | Savannah |
| Quercus douglasii Hook. & Arn. | 325524 | SJER | 0.38 | 2 | Savannah |
| Pinus sabiniana Douglas ex Douglas | 116312 | SJER | 0.14 | 3 | Savannah |
| Calocedrus decurrens (Torr.) Florin | 1957264 | SOAP | 0.65 | 1 | Conifer |
| Pinus ponderosa Lawson & C. Lawson | 626354 | SOAP | 0.21 | 2 | Conifer |
| Quercus kelloggii Newberry | 242541 | SOAP | 0.08 | 3 | Conifer |
| Quercus chrysolepis Liebm. | 198656 | SOAP | 0.07 | 4 | Conifer |
| Abies balsamea (L.) Mill. | 2390776 | STEI | 0.36 | 1 | Northern Broadleaf |
| Populus tremuloides Michx. | 1407230 | STEI | 0.21 | 2 | Northern Broadleaf |
| Tilia americana L. | 1017129 | STEI | 0.15 | 3 | Northern Broadleaf |
| Acer rubrum L. | 846965 | STEI | 0.13 | 4 | Northern Broadleaf |
| Quercus rubra L. | 721269 | STEI | 0.11 | 5 | Northern Broadleaf |
| Acer saccharum Marshall | 304397 | STEI | 0.05 | 6 | Northern Broadleaf |
| Quercus alba L. | 1128147 | TALL | 0.29 | 1 | Southern Broadleaf |
| Pinus taeda L. | 1007905 | TALL | 0.26 | 2 | Southern Broadleaf |
| Pinus palustris Mill. | 744862 | TALL | 0.19 | 3 | Southern Broadleaf |
| Liquidambar styraciflua L. | 554307 | TALL | 0.14 | 4 | Southern Broadleaf |
| Pinus echinata Mill. | 330868 | TALL | 0.09 | 5 | Southern Broadleaf |
| Liriodendron tulipifera L. | 122261 | TALL | 0.03 | 6 | Southern Broadleaf |
| Pinus contorta Douglas ex Loudon | 1238741 | TEAK | 0.34 | 1 | Conifer |
| Abies concolor (Gord. & Glend.) Lindl. ex Hildebr. | 766721 | TEAK | 0.21 | 2 | Conifer |
| Pinus jeffreyi Balf. | 520066 | TEAK | 0.14 | 3 | Conifer |
| Abies lowiana (Gordon & Glend.) A. Murray bis | 439012 | TEAK | 0.12 | 4 | Conifer |
| Abies magnifica A. Murray bis | 423231 | TEAK | 0.12 | 5 | Conifer |
| Calocedrus decurrens (Torr.) Florin | 155247 | TEAK | 0.04 | 6 | Conifer |
| Pinus lambertiana Douglas | 117623 | TEAK | 0.03 | 7 | Conifer |
| Quercus rubra L. | 1117038 | TREE | 0.16 | 1 | Northern Broadleaf |
| Abies balsamea (L.) Mill. | 1009185 | TREE | 0.14 | 2 | Northern Broadleaf |
| Populus tremuloides Michx. | 982204 | TREE | 0.14 | 3 | Northern Broadleaf |
| Betula papyrifera Marshall | 713772 | TREE | 0.10 | 4 | Northern Broadleaf |
| Picea mariana (Mill.) Britton, Sterns & Poggenb. | 572793 | TREE | 0.08 | 5 | Northern Broadleaf |
| Acer saccharum Marshall | 494667 | TREE | 0.07 | 6 | Northern Broadleaf |
| Tilia americana L. | 449669 | TREE | 0.06 | 7 | Northern Broadleaf |
| Larix laricina (Du Roi) K. Koch | 437108 | TREE | 0.06 | 8 | Northern Broadleaf |
| Pinus resinosa Aiton | 359730 | TREE | 0.05 | 9 | Northern Broadleaf |
| Thuja occidentalis L. | 288164 | TREE | 0.04 | 10 | Northern Broadleaf |
| Picea glauca (Moench) Voss | 206034 | TREE | 0.03 | 11 | Northern Broadleaf |
| Acer rubrum L. | 191871 | TREE | 0.03 | 12 | Northern Broadleaf |
| Fraxinus pennsylvanica Marshall | 125480 | TREE | 0.02 | 13 | Northern Broadleaf |
| Pinus strobus L. | 122127 | TREE | 0.02 | 14 | Northern Broadleaf |
| Tsuga canadensis (L.) Carri√re | 48679 | TREE | 0.01 | 15 | Northern Broadleaf |
| Maclura pomifera (Raf.) C.K. Schneid. | 399538 | UKFS | 0.25 | 1 | Southern Broadleaf |
| Juniperus virginiana L. | 352218 | UKFS | 0.22 | 2 | Southern Broadleaf |
| Celtis occidentalis L. | 301882 | UKFS | 0.19 | 3 | Southern Broadleaf |
| Ulmus americana L. | 163371 | UKFS | 0.10 | 4 | Southern Broadleaf |
| Quercus muehlenbergii Engelm. | 143089 | UKFS | 0.09 | 5 | Southern Broadleaf |
| Carya ovata (Mill.) K. Koch | 91871 | UKFS | 0.06 | 6 | Southern Broadleaf |
| Gleditsia triacanthos L. | 82320 | UKFS | 0.05 | 7 | Southern Broadleaf |
| Juglans nigra L. | 68406 | UKFS | 0.04 | 8 | Southern Broadleaf |
| Acer saccharum Marshall | 727435 | UNDE | 0.15 | 1 | Northern Broadleaf |
| Picea mariana (Mill.) Britton, Sterns & Poggenb. | 686836 | UNDE | 0.14 | 2 | Northern Broadleaf |
| Picea glauca (Moench) Voss | 528496 | UNDE | 0.11 | 3 | Northern Broadleaf |
| Tsuga canadensis (L.) Carri√re | 476682 | UNDE | 0.10 | 4 | Northern Broadleaf |
| Abies balsamea (L.) Mill. | 432414 | UNDE | 0.09 | 5 | Northern Broadleaf |
| Betula alleghaniensis Britton | 432101 | UNDE | 0.09 | 6 | Northern Broadleaf |
| Acer rubrum L. | 399475 | UNDE | 0.08 | 7 | Northern Broadleaf |
| Betula papyrifera Marshall | 323397 | UNDE | 0.07 | 8 | Northern Broadleaf |
| Larix laricina (Du Roi) K. Koch | 270436 | UNDE | 0.06 | 9 | Northern Broadleaf |
| Populus tremuloides Michx. | 168410 | UNDE | 0.03 | 10 | Northern Broadleaf |
| Populus grandidentata Michx. | 155654 | UNDE | 0.03 | 11 | Northern Broadleaf |
| Fraxinus nigra Marshall | 130453 | UNDE | 0.03 | 12 | Northern Broadleaf |
| Fraxinus americana L. | 89307 | UNDE | 0.02 | 13 | Northern Broadleaf |
| Pseudotsuga menziesii (Mirb.) Franco | 3987937 | WREF | 0.59 | 1 | Conifer |
| Tsuga heterophylla (Raf.) Sarg. | 1218052 | WREF | 0.18 | 2 | Conifer |
| Abies amabilis (Douglas ex Loudon) Douglas ex Forbes | 903627 | WREF | 0.13 | 3 | Conifer |
| Thuja plicata Donn ex D. Don | 664059 | WREF | 0.10 | 4 | Conifer |
| Pinus contorta Douglas ex Loudon | 3326783 | YELL | 0.74 | 1 | Conifer |
| Pseudotsuga menziesii (Mirb.) Franco | 889082 | YELL | 0.20 | 2 | Conifer |
| Populus tremuloides Michx. | 262641 | YELL | 0.06 | 3 | Conifer |

**RGB Data Experimentation**

We tested several model architectures that fuse RGB and HSI information for tree species classification. We expected that the 10cm RGB data would provide additional spatial information compared to the 1m HSI data. Data fusion is a common strategy in deep learning for remote sensing classification, the challenge is understanding at what level the data should be fused, with several reasonable options; 1) “Early”, as a stack of input data, with the HSI and RGB data resampled to the same higher resolution, 2) “Middle”, as two separate arms of a convolutional neural network with concatenation of deeply learned features during prediction, 3) “Late”, ensemble of predicted classes, either as a weighted average or a learned combination of predicted score for each tree class. We opted for the “Middle” strategy (Fig A), since fusing the “Early” layers would create enormous input data and slow models to resample the HSI data to 10cm, and the “Late” strategy was unlikely to produce high quality perfection results for the RGB data on its own.

The challenge of the “Middle” strategy is how to normalize and concatenate the two data streams. Since the 10cm RGB data is much higher resolution than the 1m HSI data, applying 2D convolutions to each input data will lead to a mismatch in feature lengths, with many more RGB features compared to HSI features. We experimented with several normalization and data reduction techniques to make these features more similar. Unfortunately, we found no evidence that the RGB data made any improvement in model accuracy (Table S2). This does not preclude that other architectures, strategies or normalization approaches won’t lead to improvement, especially when creating a unified model across sites.


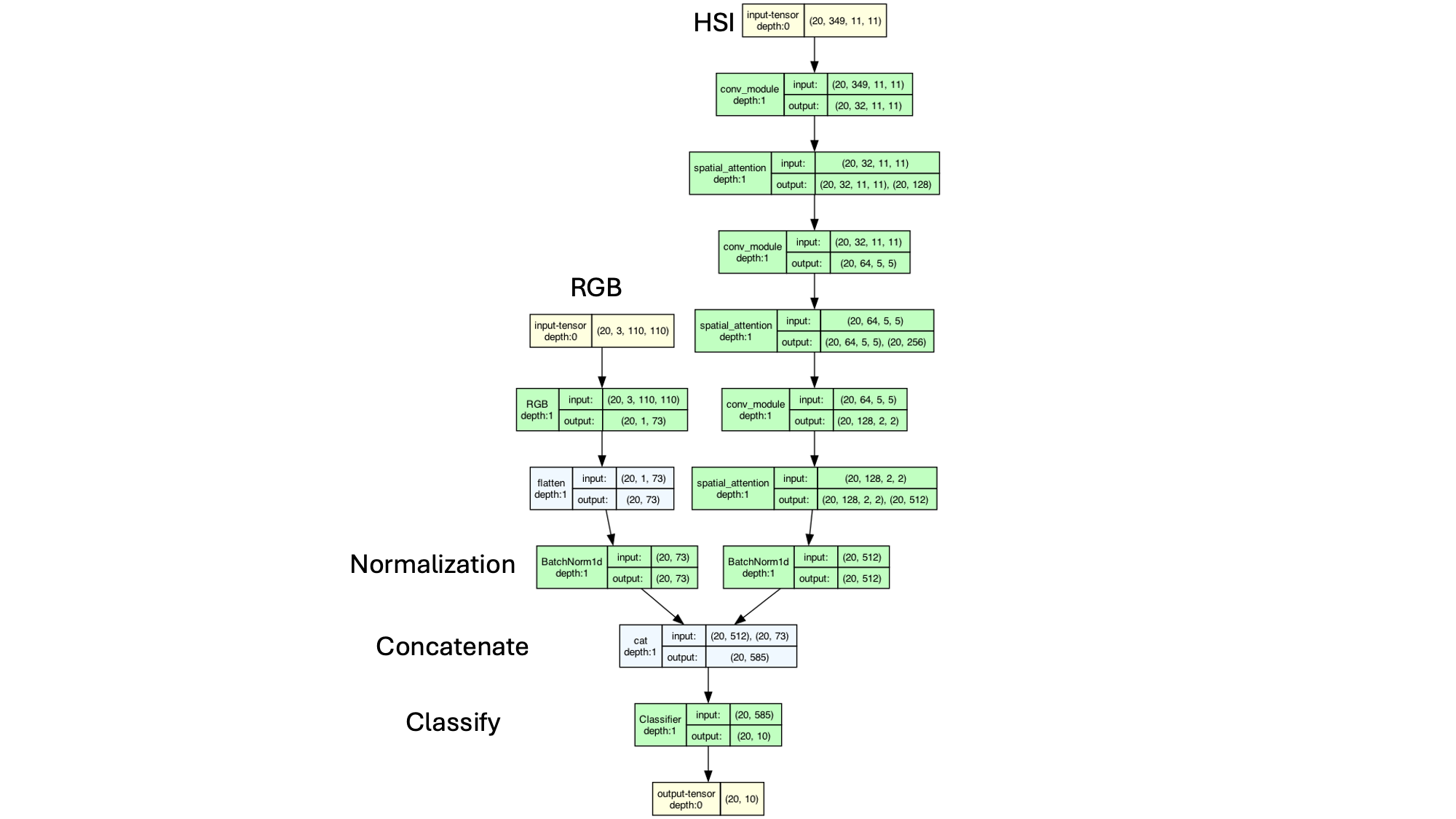


Fig A. An example model architecture for data fusion between 1m HSI data and 10cm RGB for tree species classification. In this example, a batch of crowns (n=20), each with a HSI and RGB pair, is run through the network to jointly predict tree classes (n=10). The RGB model was a resnet-50 pretrained backbone, a common RGB architecture for image-classification. The HSI architecture was the same spectral attention network used throughout the rest of the paper. The two features were min-max normalized separately before combined and a joint classifier was used to predict tree species classes.

Table B. Experiments comparing RGB, HSI and joint model for a single NEON site (OSBS). The experiments were done without the hierarchical model, or multi-temporal ensemble approaches to highlight the difference solely from source data type.

| Model Description | Micro | Macro |
| --- | --- | --- |
| HSI | 0.40 | 0.41 |
| RGB | 0.37 | 0.30 |
| RGB + HSI (Fig A) | 0.34 | 0.40 |

**NEON-wide versus per-site models**

To generate the species crown predictions, we used a separate model for each NEON site. While this yielded the best score, it greatly increased the amount of training time and reduced the portability of the workflow to new sites. Per-site models performed both better on average across all datasets, as well as at the local site level for the majority of sites (Fig B). Much of the improvement is in the rare species detection, as shown by the dramatic increase in the macro-averaged recall. For example, at OSBS, a well sampled site that we used for model validation in [27], a per-site model had a micro/macro accuracy of 0.73, 0.63 versus the test samples at OSBS in the full site scale had a micro/macro accuracy of 0.61, 0.21. This comparison should be seen as approximate, since there are 3 species not included in the per-site model that are known to occur at OSBS, but which did not meet the minimum number of test data when considering OSBS only. While we cannot know without significant further exploration, we believe that the poorer performance using a single NEON-wide model is due to three main factors: 1) combining data across sites eliminates the utility of the hierarchical approach, since it removes the logic of the initial step of modeling of the dominant class in a particular area. It is very easy to differentiate between the dominant class at OSBS (*Pinus palustris*) and the dominant species at other well-sampled sites like Harvard Forest (*Acer Saccharum*), 2) generalization across geography is complicated by environmental differences, as well as potential leaf trait variation in response to local conditions. Pooling individuals from the same species across sites may make a model that muddies the features for all sites, leading to poor performance even in well sampled areas. 3) a NEON-wide model exacerbates the class imbalance challenge within the dataset. This has a disproportionately large impact on rare species. For example, at OSBS, *Quercus hemisphaerica* co-occurs with several other difficult to identify *Quercus* species. It does not occur widely, just at two sites in the entire dataset. Despite this, the performance from the OSBS-only model drops from 76% in [27] to 37% in the single model for all NEON sites.


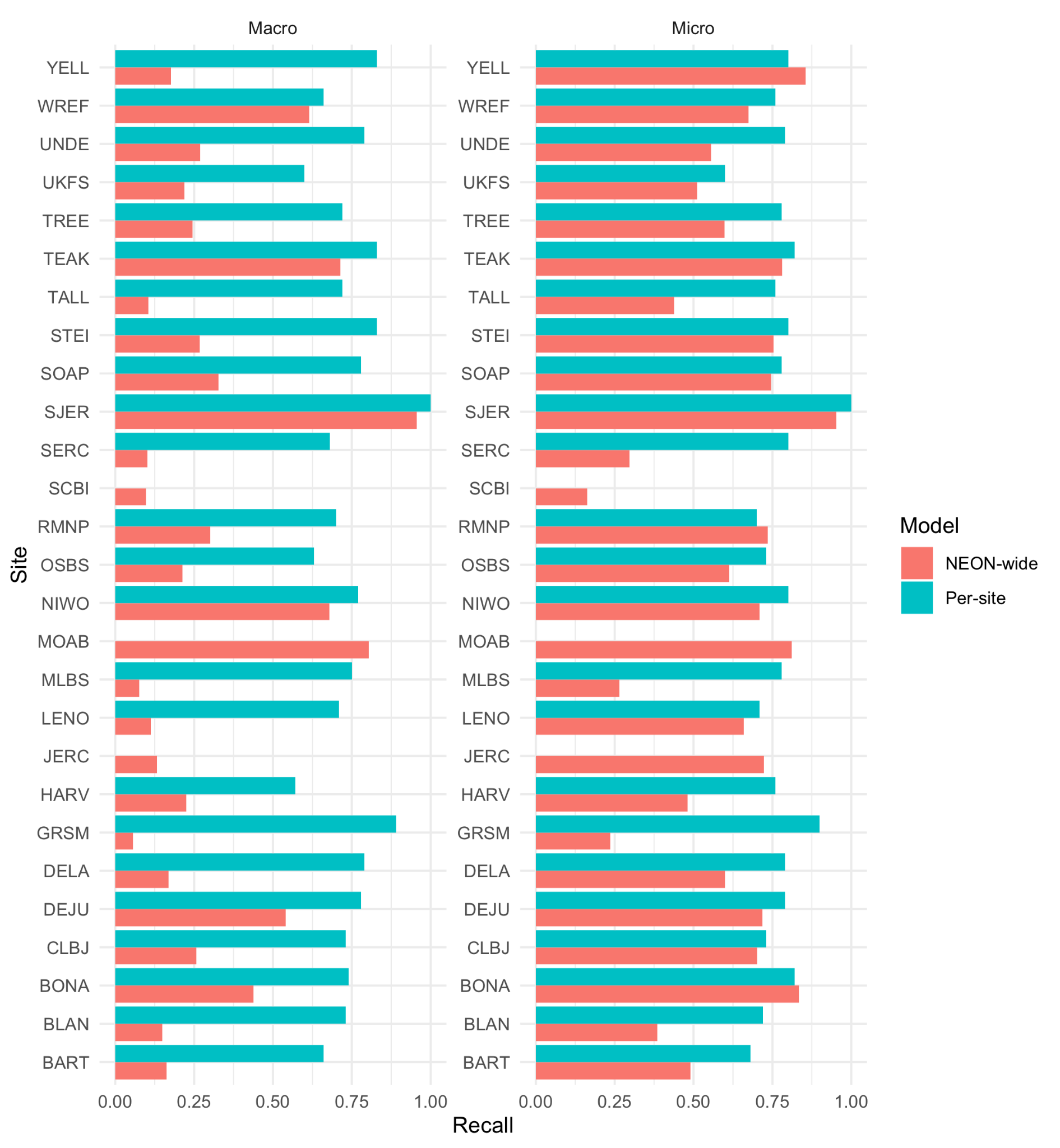


Fig B. Comparison of site-level performance for modeling workflows that use training data solely from a single site (‘per-site’) and pool training data across all sites ‘NEON-wide’. Micro averaged recall is the proportion of correctly predicted ground truth stems. Macro-averaged recall is the average recall per species, thereby weighing all species equally regardless of abundance. Several sites (JERC, MOAB, SCBI) lacked site-level predictions because the sample size per species at the individual site was too low. For the underlying data, see S4 Data.


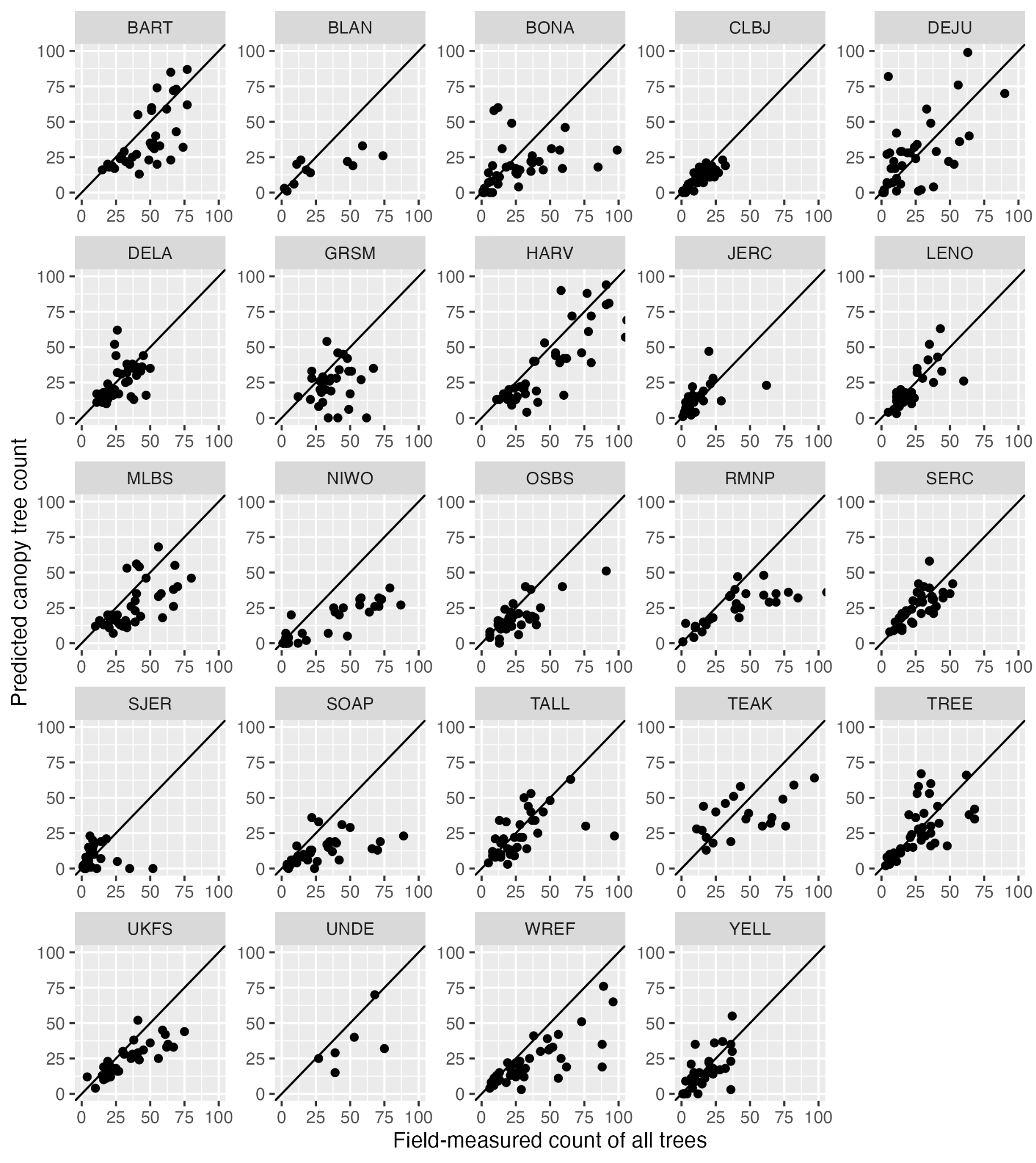
Fig C. Predicted canopy trees versus the count of all field measured trees in the NEON Woody Vegetation Structure plots. For each NEON site, the number of tree detections in the prediction data is compared to the number of field-measured detections for that NEON subplot. For the underlying data, see S5 Data.

Table C. Mean differences between predicted and observed counts, and RMSE for a generalized linear model with Poisson link function between field-measured counts of all trees and predicted canopy tree count (Fig C in S1 File).

| Site | Mean Difference | RMSE |
| --- | --- | --- |
| BART | 8.95 | 2.77 |
| BLAN | 19.08 | 4.17 |
| BONA | 16.78 | 5.78 |
| CLBJ | 3.17 | 2.65 |
| DEJU | -2.45 | 3.60 |
| DELA | 2.38 | 1.87 |
| GRSM | 13.39 | 2.97 |
| HARV | 10.95 | 2.75 |
| JERC | -1.47 | 3.17 |
| LENO | 1.48 | 1.88 |
| MLBS | 11.81 | 2.57 |
| NIWO | 17.91 | 3.31 |
| OSBS | 7.53 | 1.94 |
| RMNP | 17.07 | 3.67 |
| SERC | 1.30 | 1.79 |
| SJER | 1.74 | 3.45 |
| SOAP | 15.00 | 3.25 |
| TALL | 4.43 | 2.82 |
| TEAK | 7.50 | 3.21 |
| TREE | 1.05 | 2.69 |
| UKFS | 8.92 | 1.88 |
| UNDE | 22.86 | 3.74 |
| WREF | 13.82 | 2.94 |
| YELL | 1.21 | 2.71 |
