# Supplementary material for: Individual canopy tree species maps for the National Ecological Observatory Network
Source: PLoS Biol. 2024 Jul 16;22(7):e3002700. doi: 10.1371/journal.pbio.3002700 (PMC11251727; doi:10.1371/journal.pbio.3002700)
Supplement: Attachment — Submitted filename: PBIOLOGY-D-23-02893_R1_review - Copy.pdf [file pbio.3002700.s007.pdf]

PLOS Biology

Individual tree species maps for the National Ecological Observatory Network

--Manuscript Draft--

**Manuscript Number:**	PBIOLOGY-D-23-02893R1
**Full Title:**	Individual tree species maps for the National Ecological Observatory Network
**Short Title:**	NEON tree species maps
**Article Type:**	Initial Research Submission
**Keywords:**	Forest Ecology; Biodiversity; Deep Learning; Biogeography; Hyperspectral Reflectance; Google Earth Engine
**Abstract:**	The ecology of forest ecosystems depends on the composition of trees. Capturing fine-grained information on individual trees at broad scales allows an unprecedented view of forest ecosystems, forest restoration and responses to disturbance. To create detailed maps of tree species, airborne remote sensing can cover areas containing millions of trees at high spatial resolution. Individual tree data at wide extents promises to increase the scale of forest analysis, biogeographic research, and ecosystem monitoring without losing details on individual species composition and abundance. Computer vision using deep neural networks can convert raw sensor data into predictions of individual tree species using ground truthed data collected by field researchers. Using over 40,000 individual tree stems as training data, we create landscape-level species predictions for over 100 million individual trees for 24 sites in the National Ecological Observatory Network. Using hierarchical multi-temporal models fine-tuned for each geographic area, we produce open-source data available as 1km^2 shapefiles with individual tree species prediction, as well as crown location, crown area and height of 81 canopy tree species. Site-specific models had an average performance of 79% accuracy covering an average of six species per site, ranging from 3 to 15 species. All predictions were uploaded to Google Earth Engine to benefit the ecology community and overlay with other remote sensing assets. These data can be used to study forest macro-ecology, functional ecology, and responses to anthropogenic change.
**Additional Information:**	
**Question**	**Response**
**Competing Interests**<br><br>You are responsible for recognizing and disclosing on behalf of all authors any competing interest that could be perceived to bias their work, acknowledging all financial support and any other relevant financial or non-financial competing interests.<br><br>Do any authors of this manuscript have competing interests (as described in the PLOS Policy on Declaration and Evaluation of Competing Interests)?	The authors have declared that no competing interests exist.

Powered by Editorial Manager® and ProduXion Manager® from Aries Systems Corporation

**If yes**, please provide details about any and all competing interests in the box below. Your response should begin with this statement: *I have read the journal's policy and the authors of this manuscript have the following competing interests:*<br><br>**If no** authors have any competing interests to declare, please enter this statement in the box: "*The authors have declared that no competing interests exist.*"<br><br>* typeset<br><br>This statement is required for submission and **will appear in the published article** if the submission is accepted. Please make sure it is accurate and that any funding sources listed in your Funding Information later in the submission form are also declared in your Financial Disclosure statement.	
**Ethics Statement**<br><br>You must include an Ethics Statement in the Methods section of your manuscript if your study involved:<br>• Human participants<br>• Human specimens or tissue<br>• Vertebrate animals or cephalopods<br>• Vertebrate embryos or tissues<br>• Field research<br><br>**Not including a statement when required will delay the review process.**<br><br>General guidance is provided below. Please consult the guidelines on human subjects research and animal research for detailed instructions.	I confirm that I have read and understood PLOS' requirements for an Ethics Statement.

Powered by Editorial Manager® and ProduXion Manager® from Aries Systems Corporation

**Format for specific study types**<br><br>**Human Subject Research (involving human participants and/or tissue)**<br>• Give the name of the institutional review board or ethics committee that approved the study<br>• Include the approval number and/or a statement indicating approval of this research<br>• Indicate the form of consent obtained (written/oral) or the reason that consent was not obtained (e.g. the data were analyzed anonymously)<br><br>**Animal Research (involving vertebrate animals, embryos or tissues)**<br>• Provide the name of the Institutional Animal Care and Use Committee (IACUC) or other relevant ethics board that reviewed the study protocol, and indicate whether they approved this research or granted a formal waiver of ethical approval<br>• Include an approval number if one was obtained<br>• If the study involved *non-human primates*, add *additional details* about animal welfare and steps taken to ameliorate suffering<br><br>**Field Research**<br><br>Include the following details if this study involves the collection of plant, animal, or other materials from a natural setting:<br>• Field permit number<br>• Name of the institution or relevant body that granted permission<br><br>Please check the box to confirm your understanding of this policy, then read and agree to one of the statements.	
Please respond:<br>as follow-up to "**Ethics Statement**<br><br>You must include an Ethics Statement in	My study does not require an ethics statement.

the Methods section of your manuscript if your study involved:<br>• Human participants<br>• Human specimens or tissue<br>• Vertebrate animals or cephalopods<br>• Vertebrate embryos or tissues<br>• Field research<br><br>**Not including a statement when required will delay the review process.**<br><br>General guidance is provided below. Please consult the guidelines on human subjects research and animal research for detailed instructions.	

**Format for specific study types**<br><br>**Human Subject Research (involving human participants and/or tissue)**<br>• Give the name of the institutional review board or ethics committee that approved the study<br>• Include the approval number and/or a statement indicating approval of this research<br>• Indicate the form of consent obtained (written/oral) or the reason that consent was not obtained (e.g. the data were analyzed anonymously)<br><br>**Animal Research (involving vertebrate animals, embryos or tissues)**<br>• Provide the name of the Institutional Animal Care and Use Committee (IACUC) or other relevant ethics board that reviewed the study protocol, and indicate whether they approved this research or granted a formal waiver of ethical approval<br>• Include an approval number if one was obtained<br>• If the study involved *non-human primates*, add *additional details* about animal welfare and steps taken to ameliorate suffering<br><br>**Field Research**<br><br>Include the following details if this study involves the collection of plant, animal, or other materials from a natural setting:<br>• Field permit number<br>• Name of the institution or relevant body that granted permission<br><br>Please check the box to confirm your understanding of this policy, then read and agree to one of the statements. "	
**Data Availability**<br><br>PLOS journals require authors to make all data underlying the findings described in their manuscript fully available, without restriction and from the time of	Yes - all data are fully available without restriction

publication, with only rare exceptions to address legal and ethical concerns (see the PLOS Data Policy and FAQ for further details). When submitting a manuscript, authors must provide a Data Availability Statement that describes where the data underlying their manuscript can be found.<br><br>Your answers to the following constitute your statement about data availability and will be included with the article in the event of publication. **Please note that simply stating 'data available on request from the author' is not acceptable.** ***If, however, your data are only available upon request from the author(s), you must answer "No" to the first question below, and explain your exceptional situation in the text box provided.***<br><br>Do the authors confirm that all data underlying the findings described in their manuscript are fully available without restriction?	
Please describe where your data may be found, writing in full sentences. **Your answers should be entered into the box below and will be published in the form you provide them, if your manuscript is accepted.** If you are copying our sample text below, please ensure you replace any instances of XXX with the appropriate details.<br><br>• If your data are all contained within the paper and/or Supporting Information files, please state this in your answer below. For example, "All relevant data are within the paper and its Supporting Information files."<br>• If your data are held or will be held in a public repository, include URLs, accession numbers or DOIs. For example, "All XXX files are available from the XXX database (accession	The predictions, training data crops and shapefiles with predicted training crowns are available at https://zenodo.org/record/10041907. A csv file per site was uploaded to Google Earth engine and a public link is available as a FeatureCollection. For example, 'https://code.earthengine.google.com/?asset=users/benweinstein2010/RMNP' is the RMNP, Rocky Mountain National Park, predictions. For more on using NEON data and earth engine, see https://www.neonscience.org/resources/learning-hub/tutorials/intro-aop-gee-image-collections.

Powered by Editorial Manager® and ProduXion Manager® from Aries Systems Corporation

number(s) XXX, XXX)." If this information will only be available after acceptance, please indicate this by ticking the box below.<br>• If neither of these applies but you are able to provide details of access elsewhere, with or without limitations, please do so in the box below. For example:<br><br>"Data are available from the XXX Institutional Data Access / Ethics Committee for researchers who meet the criteria for access to confidential data."<br><br>"Data are from the XXX study whose authors may be contacted at XXX."<br><br>* typeset	
Additional data availability information:	

Powered by Editorial Manager® and ProduXion Manager® from Aries Systems Corporation

Manuscript Click here to access/download;Manuscript;PLOS_Biology.pdf

Individual tree species maps for the National Ecological Observatory Network

Short title: Individual tree species maps at NEON

Ben. G. Weinstein[1*], Sergio Marconi[1], Alina Zare[2], Stephanie A. Bohlman[3], Aditya Singh[4], Sarah J. Graves[5], Lukas Magee[3], Daniel J. Johnson[3], Sydne Record[6], Vanessa E. Rubio[7], Nathan G. Swenson[7], Philip Townsend[8], Thomas T. Veblen[9], Robert A. Andrus[9], Ethan P. White[1,10,11]

1 Department of Wildlife Ecology and Conservation, University of Florida, Gainesville, Florida, USA

2 Department of Electrical and Computer Engineering, University of Florida, Gainesville, Florida, USA

3 School of Forest, Fisheries, and Geomatics Sciences, University of Florida, Gainesville, Florida, USA.

4 Department of Agricultural & Biological Engineering, University of Florida, Gainesville, Florida, USA

5 Nelson Institute for Environmental Studies, University of Wisconsin-Madison, Madison, Wisconsin, USA

6 Department of Wildlife, Fisheries, and Conservation Biology, University of Maine, Orono, Maine, USA

7 Department of Biological Sciences, University of Notre Dame, Notre Dame, Indiana 46556 USA

8 Department of Forest & Wildlife Ecology, University of Wisconsin-Madison, Madison, Wisconsin 53706 USA

School of Environment, Washington State University, Pullman, WA

9 Department of Geography, University of Colorado, Boulder, Colorado 80309

10 Biodiversity Institute, University of Florida, Gainesville, Florida, USA

11 Informatics Institute, University of Florida, Gainesville, Florida, USA

*Corresponding Author: ben.weinstein@weecology.org

Keywords: Forest Ecology; Biodiversity; Deep Learning; Biogeography; Hyperspectral Reflectance; Google Earth Engine

Abstract

The ecology of forest ecosystems depends on the composition of trees. Capturing fine-grained information on individual trees at broad scales allows an unprecedented view of forest ecosystems, forest restoration and responses to disturbance. To create detailed maps of tree species, airborne remote sensing can cover areas containing millions of trees at high spatial resolution. Individual tree data at wide extents promises to increase the scale of forest analysis, biogeographic research, and ecosystem monitoring without losing details on individual species composition and abundance. Computer vision using deep neural networks can convert raw sensor data into predictions of individual tree species using ground truthed data collected by field researchers. Using over 40,000 individual tree stems as training data, we create landscape-level species predictions for over 100 million individual trees for 24 sites in the National Ecological Observatory Network. Using hierarchical multi-temporal models fine-tuned for each geographic area, we produce open-source data available as 1km^2 shapefiles with individual tree species prediction, as well as crown location, crown area and height of 81 canopy tree species. Site-specific models had an average performance of 79% accuracy covering an average of six species per site, ranging from 3 to 15 species. All predictions were uploaded to Google Earth Engine to benefit the ecology community and overlay with other remote sensing assets. These data can be used to study forest macro-ecology, functional ecology, and responses to anthropogenic change.

Introduction

Broadscale forest taxonomic data is essential for forest management, conservation planning, estimating carbon for global climate models and carbon offsets, and the study of cross-scale patterns of biodiversity. Historically, collection of these data has largely relied on two approaches, 1) field censused individual tree plots ranging from several dozen individuals to several thousand trees (Davies et al. 2021), which provide high quality and high resolution data, but can only be conducted over small areas for each plot; and 2) satellite-based predictions of community-level taxonomic diversity (Schäfer et al. 2016), which can be made continuously over broad scales, but lack detailed information on individual trees that is key to many aspects of forecast research and management. Individual tree predictions from high resolution image data complement these two approaches by creating a bridge between extremely high quality, but spatially and temporally restricted field data, and spatially continuous, but poor resolution data from satellite or airborne sensors. The spatial coverage of high resolution airborne imagery from planes and drones allows a more complete view of forest ecology over areas from dozens to 10,000s of hectares. Broad spatial scale individual tree predictions allow spatial patterns of species occurrence and abundance to be compared to disturbance and management regimes that naturally affect the landscape differently across spatial scales. Access to these data provides a key data collection tool to supplement high quality stand data and global satellite monitoring to facilitate the assessment of forest structure and dynamics and how they respond to ecological processes, human management and global change.

As a result of these needs, measuring individual trees using airborne sensors is becoming a key method for forest analysis and carbon calculations (Wallis et al. 2023, Tucker et al. 2023). Recent open data collection efforts by the National Ecological Observatory Network (NEON) provides an opportunity to advance our regional scale understanding of forests by providing open-access, high resolution remote sensing data over 10,000s of hectares. NEON is

a set of terrestrial and aquatic sites covering the dominant ecosystems in the United States (ranging from Puerto Rico to Alaska). Terrestrial data on tree locations, combined with annually collected airborne remote sensing data, and broad interest in conducting research at NEON sites, makes this an ideal opportunity for constructing regional scale open maps of tree species for use in ecological research.

Here, we combine airborne RGB, hyperspectral, and LiDAR data, to predict 100 million tree locations for 81 species within 24 NEON sites across the United States. Adapting the workflows developed from our research on predicting individual tree species identity from remote sensing (Marconi et al. 2022, Weinstein et al. 2023), we use a series of machine learning models to generate crown position, species label, health status and height on individual trees visible in the canopy. We make these data openly available as 1km tiles to foster research in forest ecology, as well as regional scale ecological remote sensing applications. Our work extends the dataset published in Weinstein et al. (2021) that contained crown locations, by adding predictions of species identity and alive/dead classification. The addition of species labels significantly expands the utility of this dataset to biodiversity research and natural resource management. We use a hierarchical modeling approach that also provides higher order taxonomic labels, such as 'Broadleaf' or 'Conifer' that will be useful for analyses that do not require fine taxonomic detail. In addition to providing the remote sensing derived tree maps, we also include the training and testing data used for model development to encourage further work improving the underlying computer vision models for ecological monitoring.

Each record in the dataset contains the predicted location, alive/dead classification and species identity of an individual tree based on deep learning models trained on ground truth data from human annotation of images and data collected by field researchers. While there has been significant work on many facets of the type of workflow required to generate this data, the work of creating reproducible end-to-end workflows at this scale remains underdeveloped. For example, the vast majority of research articles on tree species classification focus on a single

site, usually with a single data acquisition event. Dozens of models have been proposed, but it is unclear if they are successful when applied to a variety of ecosystems with differences in tree density, abundance distributions, and spectral backgrounds. Our goal was to apply the multi-temporal hierarchical model proposed in Weinstein et al. 2023 to sites across the United States, with a diverse set of forests including conifer, mixed hardwoods, closed canopy broadleaf and open woodlands. This model provides two key improvements over standard deep learning approaches. First, it organizes the potential classes into subgroups, allowing each model to learn better features related to distinguishing similar classes. The subgroups also allow classes that are well sampled to be separated from poorly sampled classes, thereby reducing the effect of class imbalance in favoring common species (Liu et al. 2019). Second, it combines predictions for each year of available sensor data to reduce the potential overfitting and bias due to georectification of ground-truth trees and image acquisition conditions. Using this workflow, we customize a model to each site and predict all available areas within the NEON airborne footprint that have overlapping RGB data for crown prediction and hyperspectral data for species prediction (Figure 1). These models can then be improved using targeted data collection at each site to expand species coverage and reduce misclassifications among similar species. This effort is the first open source dataset of its size and detail and is an important step in using high resolution remote sensing for cross-site, massive scale species prediction and specifically shows maturation in use of NEON airborne data from raw data collection to more refined ecological data products.

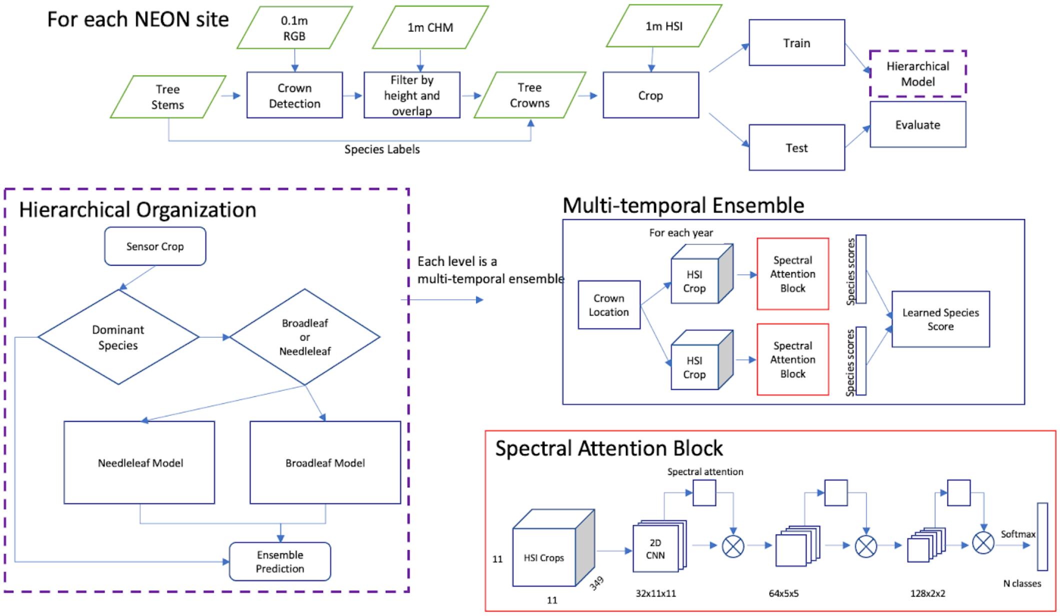

Figure 1. Conceptual workflow for species prediction at each NEON site. The figure is modified from Weinstein et al. 2023.

Materials and Methods

Workflow summary

We follow the same workflow described in Weinstein et al. 2021 for generating the crown location of overstory canopy trees using the DeepForest Python package to predict individual crown locations in the RGB camera mosaic (Weinstein et al. 2020a). DeepForest has been tested across multiple NEON sites (Weinstein et al. 2020b), with an average accuracy of over 70% between predicted and hand-annotated tree crowns. Tree crowns with less than 3m maximum height in the LiDAR derived canopy height model are removed. At this stage in the workflow each individual tree has a unique ID, predicted crown location, crown area and confidence score from the DeepForest tree detection model.

Following individual tree detection, we classify each individual's health status as Alive or Dead based on its appearance in the RGB data. Since NEON captures airborne data during the leaf-on season, any standing tree with no leaf cover was annotated as 'dead'. This label should be interpreted as provisional since trees can lose leaves due to a variety of causes such as insect defoliation in one year, but ultimately recover over time. Presented in Weinstein et al (2023), this Alive-Dead model is a two class resnet-50 deep learning neural network trained on hand-annotated images from across all NEON sites. During prediction, the location of each predicted crown is cropped and passed to the Alive-Dead model for labeling as Alive (0) or Dead (1) with a confidence score for each class.

To classify each tree crown to species, we use a multi-temporal hierarchical model (Weinstein et al. 2023). The predicted species label confidence score, as well as labels from the higher taxonomic levels, are included in the shapefile (Table 1).

Table 1. Data available for each predicted crown. Crowns are organized into 1 km shapefiles with UTM projection and follow the naming scheme from NEON's AOP data, with a geographic index at the top left corner. For sites with fewer than 5 species, the broadleaf and conifer labels are not available, as they are largely redundant with the species present and were all modeled jointly.

Column Name	Definition
Geometry	A four pointed bounding box location in utm coordinates.
indiv_id	A unique crown identifier that combines the year, site and geoindex of the NEON airborne tile (e.g. 732000_4707000). The utm coordinate is the northwest corner of the tile.
sci_name	The full latin name of predicted species aligned with NEON's taxonomic nomenclature.
ens_score	The confidence score of the species prediction. This score is the output of the multi-temporal model for the ensemble hierarchical model.
bleaf_taxa	Highest predicted category for the broadleaf model

bleaf_score	The confidence score for the broadleaf taxa submodel
oak_taxa	Highest predicted category for the oak model
dead_label	A two class alive/dead classification based on the RGB data. 0=Alive/1=Dead.
dead_score	The confidence score of the Alive/Dead prediction.
site_id	The four letter code for the NEON site. See https://www.neonscience.org/field-sites/explore-field-sites for site locations.
conif_taxa	Highest predicted category for the conifer model
conif_score	The confidence score for the conifer taxa submodel
dom_taxa	Highest predicted category for the dominant taxa mode submodel
dom_score	The confidence score for the dominant taxa submodel

Airborne Sensor Data

The NEON airborne observation platform (AOP) collects remote sensing data on an annual basis during leaf-on conditions for all sites. We used four NEON data products 1) orthorectified Camera Mosaic ('RGB' NEON ID: DP3.30010.001), 2) ecosystem Structure ('Canopy Height Model' NEON ID: DP3.30015.001), 3) hyperspectral surface reflectance ('HSI' NEON ID: DP1.30006.001), and 4) vegetation structure (NEON ID: DP1.10098.001) (National Ecological Observatory Network (NEON) 2021). All data were downloaded in August 2022 and were the RELEASE form (NEON 2023). The 10 cm RGB data were used to predict tree crown locations necessary for associating field labels and sensor data during model development. RGB data were also used to identify dead trees during our prediction workflow. The 1 m canopy-height model was used to determine which field collected data were likely to be visible from the air, as well as to define a 3 m minimum tree height threshold during the prediction workflow. The HSI data is used to differentiate tree species based on spectral reflectance. The HSI data spanned approximately 420-2500 nm with a spectral sampling interval of 5 nm producing a total of 426 bands. NEON provides orthorectified images with a pixel size of 1 m^2 in 1 km^2 tiles that are

georectified and aligned with the RGB and Canopy-Height-Model. For more information on hyperspectral data processing and calibration see NEON technical document NEON.DOC.001288.

Field-based species labels

The NEON Vegetation Structure dataset is a collection of tree stem points within fixed-area field plots; plot locations are allocated across sites according to a stratified random, spatially balanced design (Barnett et al. 2019). All trees in sampled areas with a stem diameter > 10 cm are mapped and measured for diameter, height, health status, and species identity. Building on this NEON dataset, we contacted researchers at each NEON site to find as many stems as possible outside the NEON woody vegetation sampling plots. We collected 22,072 additional canopy trees from a variety of sources, including several large ForestGEO plots co-located at NEON sites (Davies et al. 2021) and public data (Veblen et al. 2021). We followed the taxonomic hierarchy used by NEON with the exception of genus-only, subspecies and variety labels. To connect the species information from the ground-based stem points with the airborne sensor data, we adopted a heuristic approach for filtering data (Figure 2). We began with raw stem data for 41,036 individuals. The first step is to remove stems that are dead or broken, do not have a species label, or are less than 3m in field measured height. Whenever dbh is available, stems less than 10cm were discarded. After this step there were 40,883 stem points remaining. We then compare the field-measured height to the height of the LiDAR-derived canopy model for the closest available year. If the difference between the LiDAR derived and field height was more than 4m, we discarded the tree stem. After this step there were 38,173 stem points remaining. We then overlaid these height-filtered points to crown bounding box predictions made from the DeepForest RGB algorithm. If more than one height-filtered point fell within the predicted canopy crown box, we selected the tallest point using the canopy height model since this was most likely to be the dominant tree in the canopy. The shorter tree stems

that overlapped the bounding box were discarded. If a point did not overlap with any bounding box, we created a 1m buffer around the point to serve as a crown box. We refer to these crowns as ‘fixed boxes’ and these were allowed only to be included in training data, but never in testing data due to lower confidence in associating species labels and sensor pixels. After this step, there were 31,736 points remaining to be used for model training and validation.

To assess the proportion of species known to occur at each site captured by the filtered data we compared the final species list with a reference data set of the original data. The original data was filtered by a minimum field-reported height of 3m and a minimum of 2 sampled points to ensure comparison only among canopy species. While this number captures the proportion of biodiversity at the site captured by the model, it does not account for the strong imbalance in species abundance, in which a small number of species account for a large proportion of trees in an area. Therefore, we also calculated the proportion of tree samples for each species in the final model. For example, if we had 100 samples in a geographic site in the original data set, with 97 samples coming from species A and 3 samples from species B, and the final model only contained species A, the proportion of species covered would be 0.5, but the proportion of stems would be 0.97.

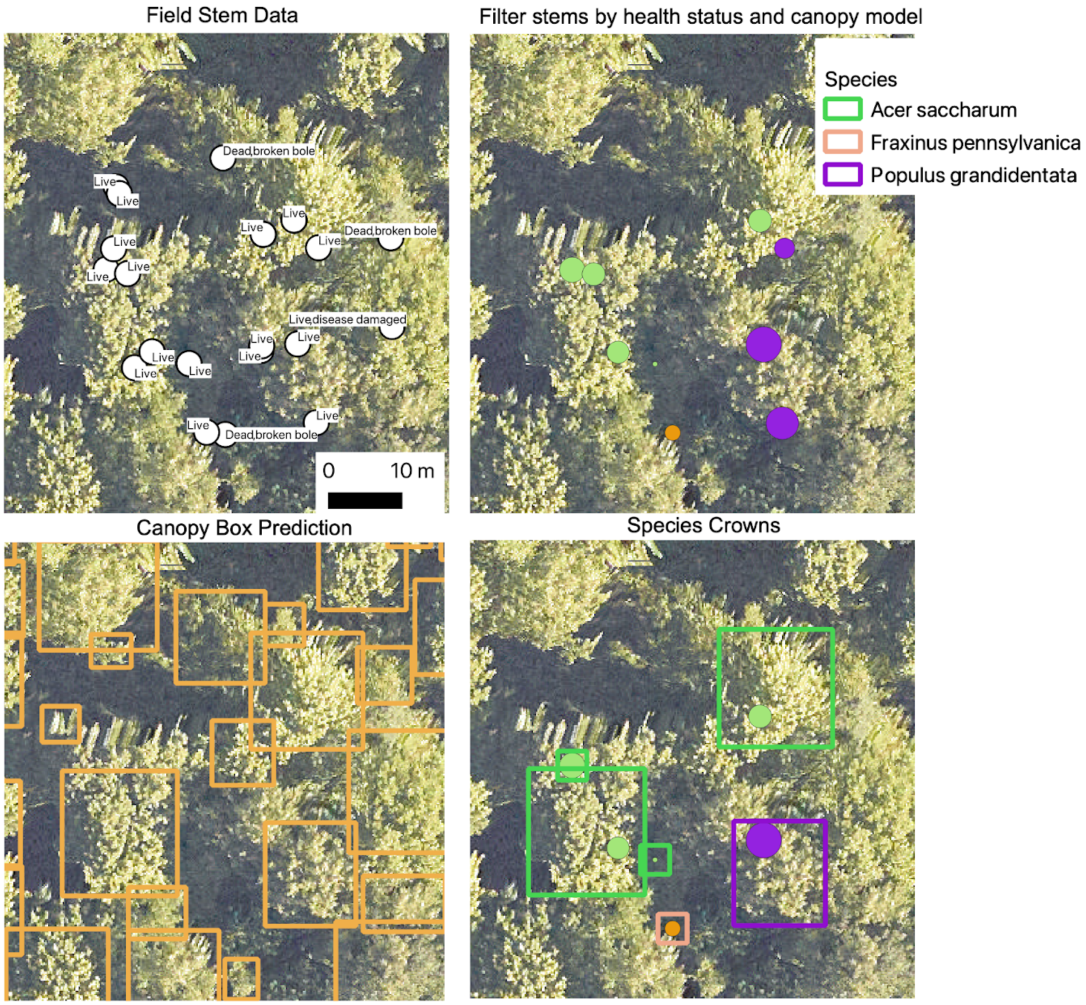

Figure 2. Example workflow for filtering stem data to associate with crown pixel area. Size of the dots in panels b and d are proportional to the stem DBH.

Multi-temporal Hierarchical Species Prediction

The species prediction model is a hierarchical collection of submodels, which divide classes into smaller categories. The top model predicts ‘Broadleaf’, ‘Conifer’ and optionally the dominant tree species class. A species was considered dominant if it consisted of more than 40% of the training data. This approach allows rare classes to be aggregated and separated from the dominant class. Without this or another approach to reduce class imbalance, common machine learning approaches will predict most samples as the dominant class regardless of spectral

signal. After prediction in the first subgroup, samples that are predicted as ‘Broadleaf’ are then passed to the Broadleaf submodule, and samples that are predicted as ‘Conifer’ are then passed to the Conifer submodule. This structure was maintained for the majority of sites, but we did allow some site specific customization. For example at the OSBS site, the many similar oak species were split off into their own oak submodule within the broadleaf submodule. This ‘mixture-of-experts’ approach is common in computer vision, but comes at the cost of training complexity and time.

Each submodule consists of a 2D spectral attention block (Figure 1) with 3 convolutional layers and a max pooling spectral attention layer following Hang et al. (2021). Batch normalization is used to normalize layer weights after each convolution. This spectral attention block is then repeated for each year of airborne sensor data to create an ensemble model. For example, if there are four years of available hyperspectral data for a geographic location, we predict four classification outputs and then combine them to create the final prediction. This is based on the assumption that the canopy trees at each geographic location are unlikely to change species label among years at short time scales. This form of multiple view modeling has been used elsewhere in airborne ecological modeling and in the wider computer vision community. A weighted average among all years is used to create the sample prediction for each crown. This relative weight among years is a learned parameter for each submodel. For more details and validation, refer to Weinstein et al. 2023, which showed improved performance for common and rare species accuracy compared to a flat single year model.

To train species classification models, we opted to build a different model for each NEON site. While this limits the portability of these models to new locations, our aim was to build the best prediction map for each geographic area. To help reduce overfitting on a small number of samples per site, but to maximize site-specific features, we adopted a pretraining strategy to use data from all sites, but only include the species at the focal site, followed by fine-tuning on samples only at the target site. This was repeated for each site. The combination of global and

local data was shown to outperform local-only models in Marconi et al. 2022 for a portion of this dataset. For each site we pretrained for 200 epochs decreasing the learning rate of each submodel based on performance on the focal site test data. We then fine-tuned this model with the available annotations at the target site for 200 epochs. The learning rates differed among submodules, with the dominant class and conifer submodules having an initial learning rate of 10e-5, and the broadleaf model starting at 10e-4. We allowed batch size to vary between 12 and 24 depending on the site to account for differences in class imbalance and dataset size.

To determine the evaluation accuracy of species predictions, we developed a train-test split with a minimum of 10 samples per class. To minimize the potential effect of spatial autocorrelation in hyperspectral signature between training and test datasets, we adopted a spatial block approach. All samples within a NEON plot, or within a 40m grid for the non-NEON contributed data were assigned to training or test. We performed this assignment iteratively until the minimum number of samples per class were in the test dataset. The remaining samples were used to train the model. If there were multiple ways to keep as many species as possible while maintaining the spatial design, we selected the training-test split with more species in the test dataset. For each site we evaluated the accuracy and precision of each species. To get the site-level score, we used both micro-averaged accuracy and macro-averaged accuracy. Micro-averaging weights all samples the same, and therefore is largely driven by the performance of the common species. Macro-average weights all species the same, giving greater importance to the rare species as compared to their frequency in the dataset. We also computed the accuracy of the higher order taxonomic labels (e.g. ‘Broadleaf’ vs ‘Conifer’), which may be useful to downstream applications in which coarser categories are sufficient.

Results

We developed individual tree species models for 81 species at 24 NEON sites (Table 2). After filtering the data for canopy heights, and visible crown prediction, there was an average of 6.56 species per site, with a maximum of 15 species (HARV) and minimum of 3 (DEJU and SJER). Compared to reference species lists filtered for canopy species, the crown data set covered 48.2% of the total species diversity known to occur at the sites (Figure 3). These species account for an average of 85.4% of the stems in the NEON sites. The average model had a micro-averaged accuracy of 78.8% and a macro-accuracy of 75.8% (Table 2). Sites with more data generally performed well, with a general pattern of decreasing species-level accuracy with fewer data (Figure 4). Consistent with previous work, the highest performing sites, including TEAK, NIWO, and YELL, were dominated by conifers and had relatively low species diversity (Marconi et al 2022). Models performed more poorly in southern broadleaf forests, such TALL and SERC, with higher biodiversity, closed canopy structure, and/or low data coverage per species (Marconi et al 2022). The most abundant species at a site typically had the highest accuracy, with lower accuracy for rarer species (Figure 4).

Applying the best model for each site to all available airborne tiles, we predicted 100,021,471 trees with an average of 3.56 million trees per site. Of the 24 sites, 17 are heavily forested with near continuous canopy cover. These sites tend to have high species diversity at local scales with overlapping crown boundaries (Figure 5). The remaining sites cover savannah and other open forest types with lower species diversity and more open canopy structure. Patterns of biodiversity are highly scale dependent with grouping of similar species in local areas, with complex patterns of species patches at broader scales within the same site (Figure 6). Ranking the predicted species abundance for each site, there is a pattern of the most commonly predicted species containing approximately 60% of crowns (Figure 7). The dominant species was slightly less abundant in the southern broadleaf sites with 30% to 40% of crowns

belonging to the most commonly predicted species. The predicted abundance curve drops sharply with the 5th most common species at 10% of crowns at the majority of sites. Viewing the predictions at the largest spatial extents, there is a broad range of species presence patterns, with some sites showing highly mixed species to other sites with distinct autocorrelation and species patterns at all spatial scales (Figure 8). In many cases there are noticeable patterns in species occurrence along environmental and topographic gradients.

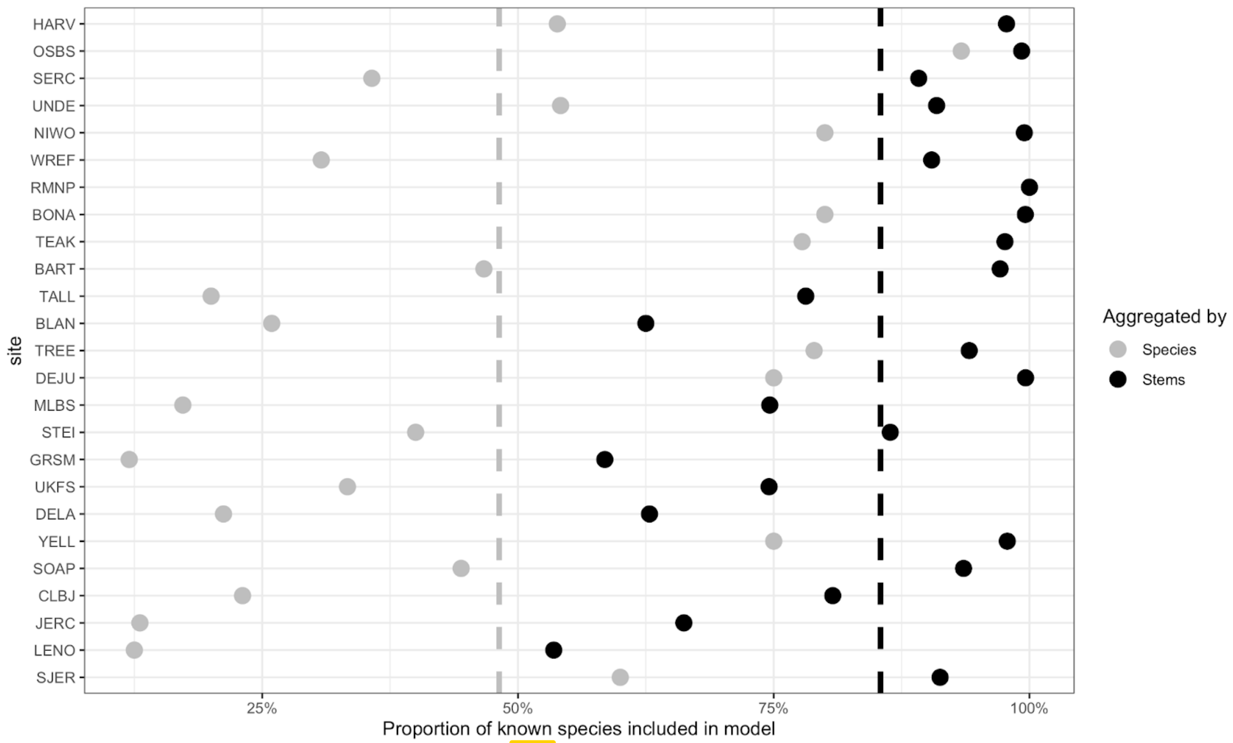

Figure 3. The proportion of species and stems included in the model for each site compared to all available stem points. For example, 49% of species known to occur at BART were included in the model, but these species capture over 90% of the sampled trees at the site. The raw data have been filtered for canopy species based on field measured height and at least 2 field points. These filters avoid comparison with tree species that are not visible from above and potential data entry errors or rare species misclassifications. The sites are ordered by the highest number of raw samples in HARV (n=15,262), to the lowest number of raw samples in SJER (n=103). For a complete list of each species in the model and the canopy-filtered data, see Table S1. The dashed line is the mean number of species across sites for both species and stem proportions.

298 Table 2. Evaluation scores for each NEON site included in the dataset.

Site	State	Micro Accuracy	Macro Accuracy	Species	Train Samples	Test Samples
SJER	CA	1.00	1.00	3	47	27
GRSM	NC	0.90	0.89	3	200	29
TEAK	CA	0.82	0.83	7	713	67
BONA	AK	0.82	0.74	4	584	103
STEI	MI	0.80	0.83	6	283	82
NIWO	CO	0.80	0.77	4	852	46
YELL	WY	0.80	0.83	3	390	10
SERC	MA	0.80	0.68	11	816	287
DELA	AL	0.79	0.79	7	166	72
DEJU	AK	0.79	0.78	3	571	52
UNDE	MI/WI	0.79	0.79	13	547	178
SOAP	CA	0.78	0.78	4	223	37
MLBS	VA	0.78	0.75	5	363	54
TREE	WI	0.78	0.72	15	643	168
WREF	WA	0.76	0.66	4	598	97
TALL	AL	0.76	0.72	6	250	125
HARV	MA	0.76	0.57	15	9782	1194
OSBS	FL	0.73	0.63	14	3293	240
CLBJ	TX	0.73	0.73	3	187	30
BLAN	VA	0.72	0.73	8	271	79
LENO	AL	0.71	0.71	3	74	28
RMNP	CO	0.70	0.70	7	671	99
BART	VT	0.68	0.66	7	514	125
UKFS	KT	0.60	0.60	8	204	85

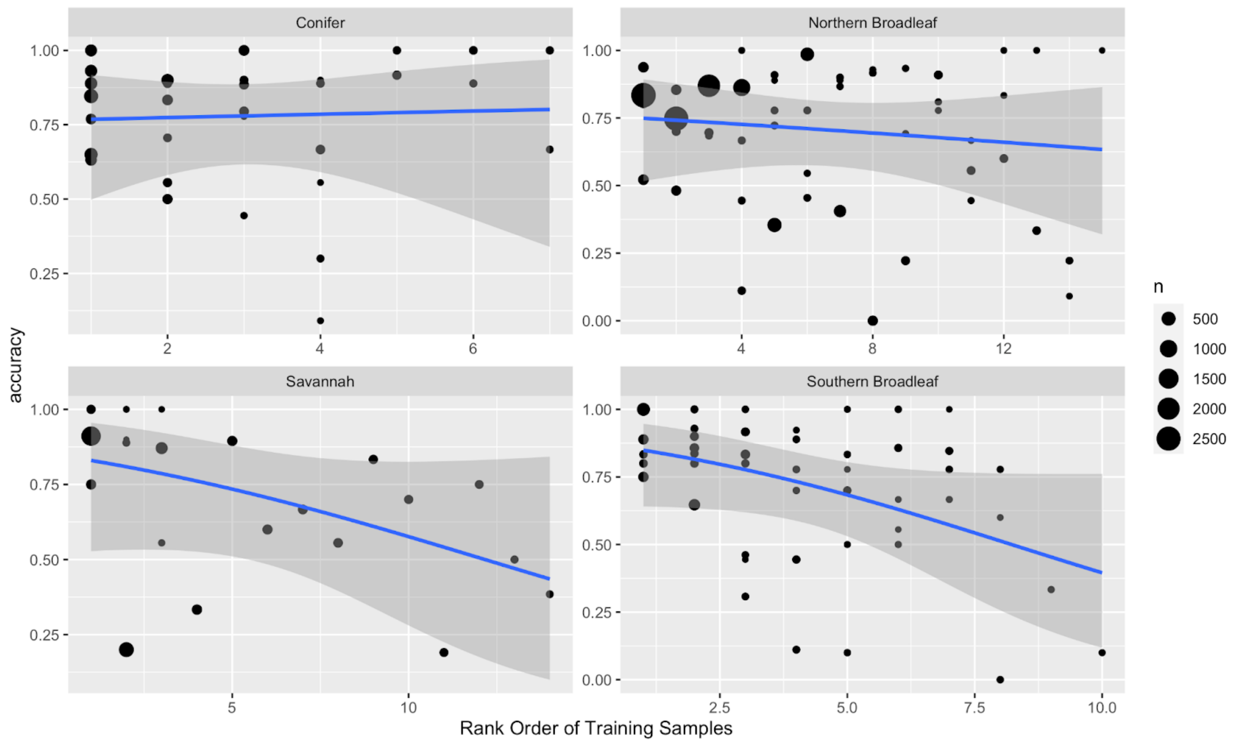

Figure 4. Rank order abundance and evaluation accuracy for each species for each geographic site. A binomial model was fit for each habitat to relate the rank order abundance of each species and evaluation accuracy. Each point is one species within each NEON site model.

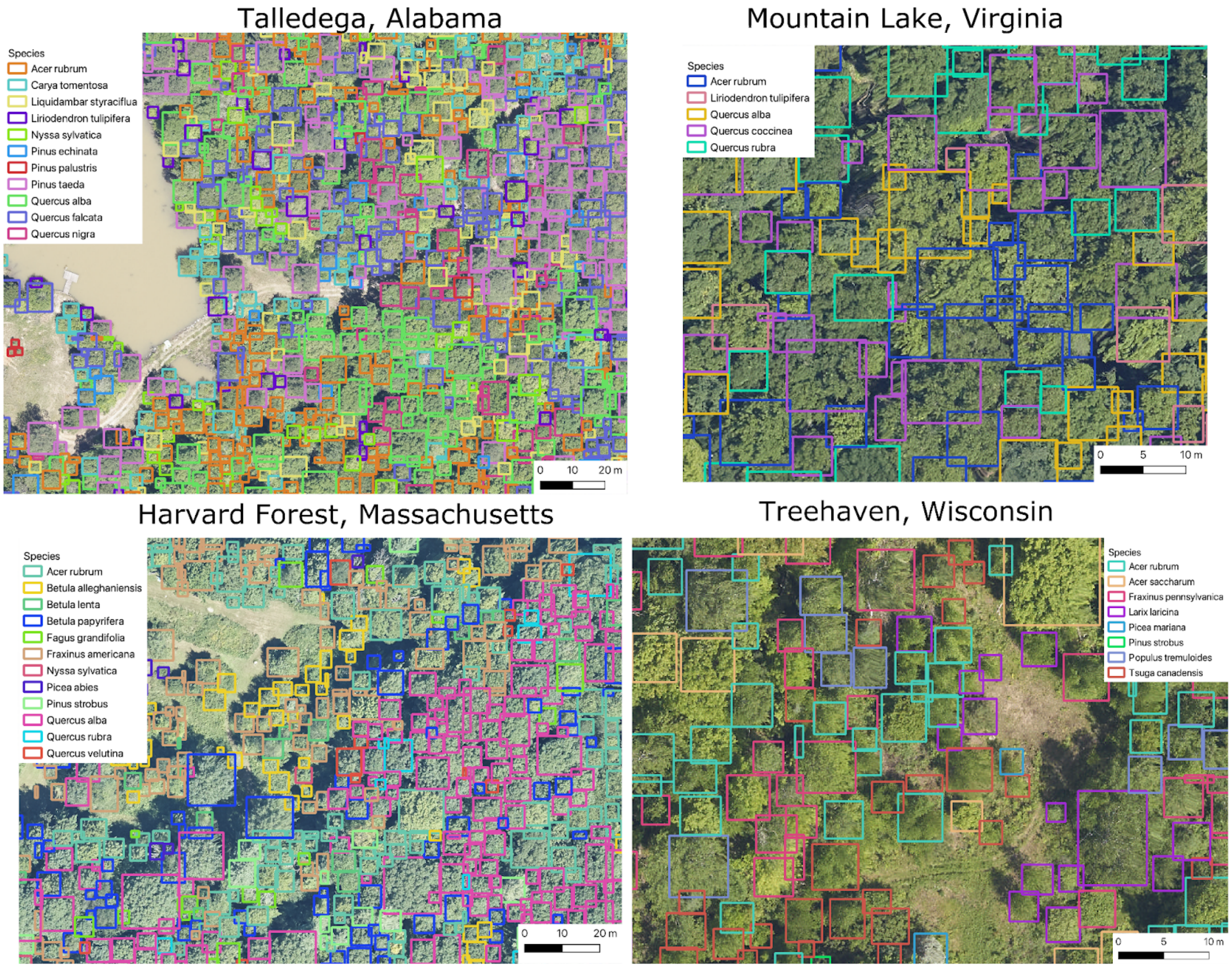

Figure 5. Example tree detections and species labels for four NEON sites with closed canopy deciduous forests.

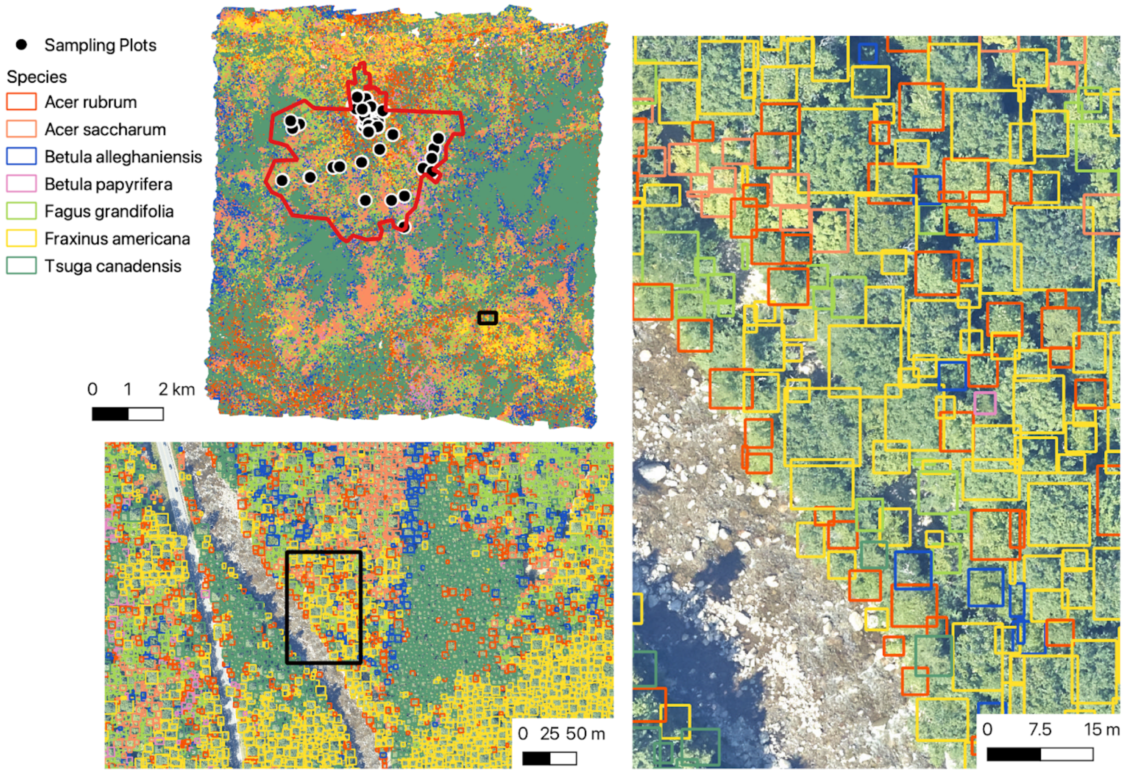

Figure 6. Overview from BART showing 5,110,326 tree predictions for 7 species at three spatial scales. The location of NEON sampling plots and the boundaries of the Bartlett Experimental Forest are shown in the largest scale.

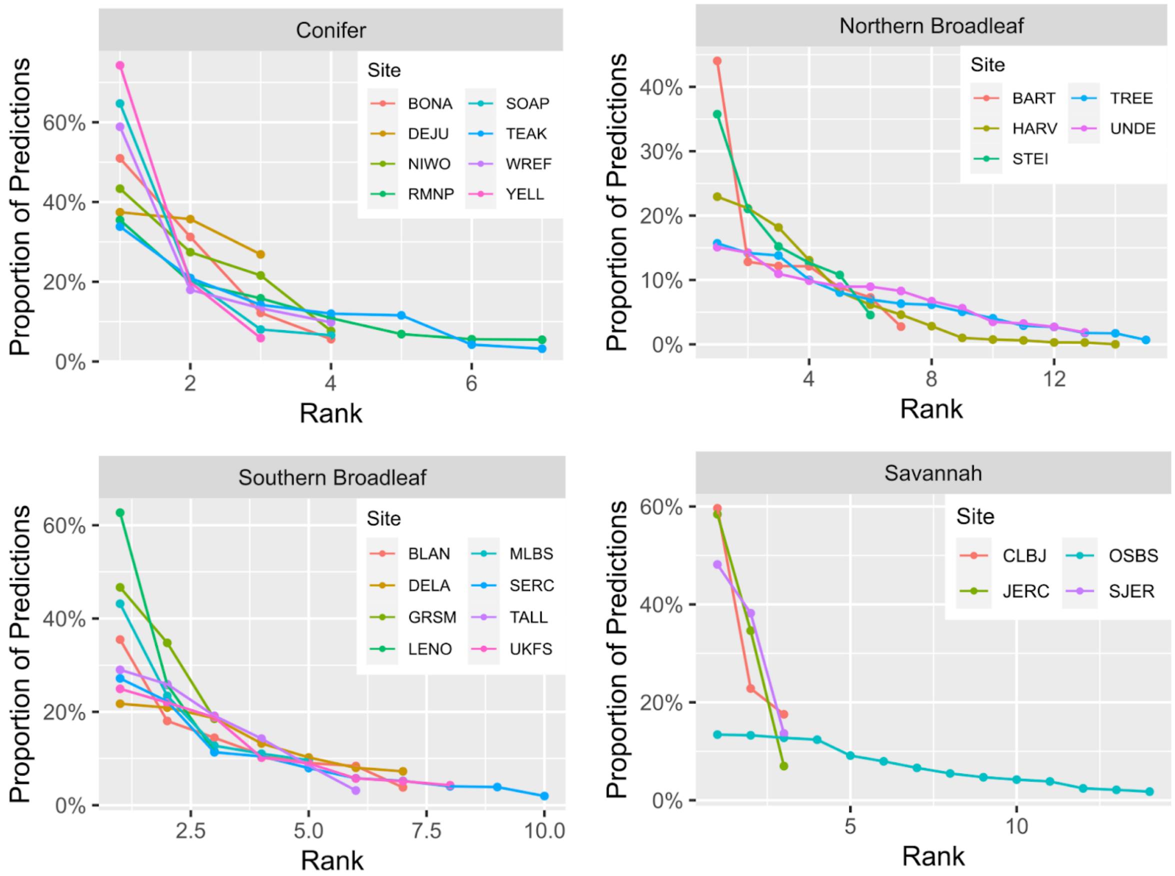

310

311 Figure 7. Rank order abundance for the predicted crown species labels for each site. Each point

312 represents a species predicted at a site. For species identity and totals per site see Table S1.

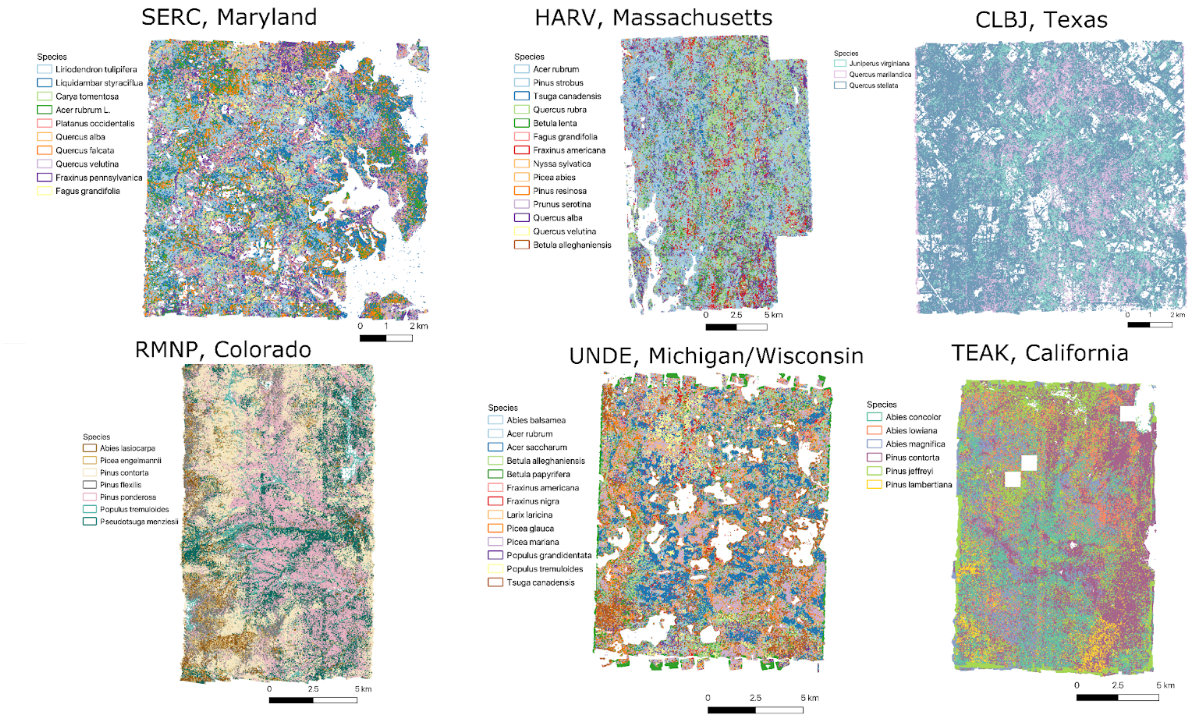

Figure 8. Overview of multiple sites spanning a broad range of forest types in the dataset.

Discussion

The goal of this work was to produce individual-level maps of tree species for sites across the National Ecological Observatory Network (NEON) by applying a previously developed hierarchical multi-temporal model applied to high resolution hyperspectral data. The maps provide the foundation for cross-scale research on forest macrosystems biology as well as site-specific questions on biodiversity, tree health, forest demographics, and wildlife habitat. We produced maps covering tens of millions of individuals with the available data for each site. While there remains significant opportunities to improve each portion of this workflow, this dataset is an important step towards fine-grained, tree-level ecological information at landscape scales and continental extents. We hope that the publication of this dataset will spur both research on the ecology of forest ecosystems as well as improvements in computer vision to refine the many challenging obstacles in assembling wall-to-wall tree maps at massive scales.

By repeating a general model architecture across a broad array of sites, we identify trends in model performance as a function of forest type. These patterns are: 1) decreased accuracy with an increasing number of species, 2) higher accuracy at sites with more open canopy structure, and 3) a general tendency of higher performance for conifer over broadleaf species. While these patterns are well known from individual site studies (e.g. Maschler et al. 2018, Hastings et al. 2020), we can begin to quantify their effects by comparing a general modeling approach across the 24 sites. In general, the most common species can achieve roughly 75 to 85% accuracy at well sampled diverse sites. There was strong geographic bias even among sites with similar habitats. Northern broadleaf sites in general had better accuracy than the more diverse southern broadleaf sites. This is likely, in part, due to the higher tree diversity in the southern states. Models with more species often have lower accuracy because optimizing complex spaces is harder with more tradeoffs among species. The model itself is larger with more parameters and more prone to overfitting to the small amounts of data

available for less common species. Increased species diversity also increases the chance for neighboring trees to be of different species and pollute crown edge pixels with spectra of a different neighboring species and confuse the training and test labels.

The level of spatial heterogeneity in habitats and species turnover may also affect model performance. High local turnover will make the training data less representative of the total biodiversity, as well as the spectral background in which species occur. The ‘Northern Broadleaf’ forests appear to be more admixed than ‘Southern Broadleaf’ forests, or atleast the number of unique habitats are more well sampled by NEON’s terrestrial plot design (Barnett et al. 2019). Both ‘savannah’ and ‘conifer’ sites appear to have relatively low species diversity as well as high local mixture in species, making sampling more tractable. Explicit measures of spatial autocorrelation in species presence will be valuable in understanding the ecology of these ecosystems, as well designing surveys for collecting data for species classification.

Despite the growth in the number of articles focusing on individual tree segmentation, individual tree health status, and individual tree species, the task of designing end to end predictive workflows is underdeveloped. Focusing on the full workflow highlights challenges for real-world applications that are typically missed when focusing primarily on model development. For example, many published analyses use the location of known tree stems to anchor the location of evaluation points. This makes sense, as it disentangles the lower accuracy introduced by incorrectly associating species labels with tree crowns from the performance of the species model. However, from the point of view of downstream applications, separating the classification error and detection error in this way will make it appear that the expected accuracy of predictions is higher than it will be when making predictions for trees across the full landscape. We addressed this by mirroring what would happen in an end-to-end predictive workflow. We use our crown detection algorithm to detect crowns in the region of evaluation points and then match a bounding box with each evaluation tree based on distance to centroid and predicted CHM height. Our approach more closely mimics the prediction environment

during evaluation and should therefore provide more accurate characterization of our confidence in predictions for different species. Judging future model developments from the perspective of future users is key in moving beyond the concepts of accuracy and utility for broad scale fine-grained species predictions.

Focusing on optimal end-to-end predictive workflows also highlights the importance of viewing these data as the first step in an ongoing iterative process for generating the best possible characterization of canopy tree biodiversity across increasingly larger areas. The largest obstacle to improving model accuracy is the availability of training data. As additional data sources are available, the models can be updated to include new species and existing species in different ecological contexts. We have found that targeted data sampling can yield 10 to 20% improvements in accuracy, and significantly broaden the species list, with only a few days or weeks of field work (Box 1). The simplest form of data needed is a geospatial point of a tree stem and its species label. Since the label refers to the entire crown, the geospatial point needs to be accurate, but only needs to be precise enough to ensure that it falls within the associated crown box predicted by the detection algorithm. Therefore, while standard GNSS units with meter level accuracy are optimal, handheld GPS may be sufficient. This makes the most pressing challenge for additional data collection deciding which trees to sample. Given millions of trees in a forest, it is important to use field sampling efforts efficiently to improve model scores. Collecting data on the common species will have limited impact on model performance, as it will increase the class imbalance and only minimally improve common species accuracy.

While new quantitative approaches are needed to simplify the process of collecting targeted field data, we can describe three general strategies that can be used together 1) Use expert knowledge of the site to identify areas containing species that are underrepresented in the training set. This strategy is most helpful for sites with habitat heterogeneity (e.g., differences in bedrock geology, areas with different successional trajectories) and areas of

known underrepresented species are spatially restricted. 2) Using the model confusion matrix and predictions from the initial model, target sampling of the species with unexpected confusion patterns. Using the confusion matrix focuses directly on species where the model performs poorly and using initial predictions to identify the location of less common species helps reduce the time and cost of sampling less common trees. Unexpected patterns of confusion, in which two underrepresented species, which are not visually similar are confused by the model, may be an indicator that the crowns of these species in the training data set include parts of neighboring crowns, thus they are mingled with spectra from neighboring trees. This can potentially be quickly improved on with additional data on those species. 3) Use the confidence scores of the species predictions to identify tree species that are either known and poorly captured by the model or that may be new species not currently included in the model. Machine learning models typically lack the ability to skip making a prediction for some samples where the match to known labels is low, and therefore must assign each crown to one of the training classes. While the confidence scores are not calibrated, and therefore should not be seen as true probabilities, they are generally helpful for estimating the likelihood of a sample being correctly assigned a species label. For samples that are assigned to common classes, but have low confidence, this may be an indication of less common species (Box 1 Figure 2). Finding these highly confused samples may have strong leverage on model performance.

As improvements are made through new data collection and other improvements to workflow, predictions from these large scale mapping efforts should be continually updated. This requires the development of end-to-end remote sensing workflows that are reproducible and scalable. Currently our workflow for generating predictions for a full NEON site, including ingesting data, fitting the model, and generating predictions, can be rerun with a single command in 8 hours on 1 GPUs, allowing us to quickly incorporate additional data to yield improved maps. Our goal is to work with researchers near individual NEON sites to quickly collect new field data that allows us to produce improved biodiversity maps for each site,

building on our work at OSBS (Weinstein et al. 2023) and our work with N. Swenson on UNDE (Box 1). This will ideally result in an iterative process where improved biodiversity data and maps are generated, local experts use those improved maps and model results to identify areas for further improvement, new data is collected, and the cycle continues until data and maps are sufficiently accurate for the biological questions of interest.

Box 1. In-depth examination of collecting new data to improve models.

To increase the species coverage and accuracy of these models, we need additional data collection at each NEON site collected outside of the NEON field sampling plots. We encourage researchers at each site to submit data to improve models. In (Weinstein et al. 2023), we outline multiple weeks of sampling for specific habitats at the OSBS field site. We were able to improve species coverage and better differentiate similar oak species after collecting several hundred targeted samples. Here, we outline one effort by N.G. Swenson and V.E. Rubio to improve the model at UNDE through a series of short afternoons of targeted data collection (Box 1; Figure 1). The original model has an accuracy of 67.8% micro-accuracy, 61.6% macro-accuracy covering 12 species. Overlaying the predictions over a recently mapped forestry plot, three areas of need were identified, 1) several key species were missing from current predictions, 2) there was overprediction of *Fraxinus nigra* compared to the abundance expected by field researchers, 3) there was high confusion between two closely related *Populus* species. Using these goals to target trees, data were collected along easy to access roads and forest edges. It is unknown whether the spectral signatures of these samples are representative of the same species in dense forest. However, the speed of data collection in these areas may compensate for some intraspecific variation introduced by either background or altered growth characteristics of trees along roads and edges. A total of 157 stems of twelve species were collected, though several consisted of only one sample per species.

After training the model on the additional stems, the micro-averaged accuracy increased from 67.8% to 77.7% and the macro-averaged accuracy increased from 61.6% to 79.1% while adding 1 additional species to the test dataset. The accuracies of the two closely related *Populus* species increased from 66% and 54% to 72% and 82%, respectively. While follow up surveys did show the presence of *F. nigra* in the area at higher than expected amounts, we continue to see overprediction of *F. nigra*.

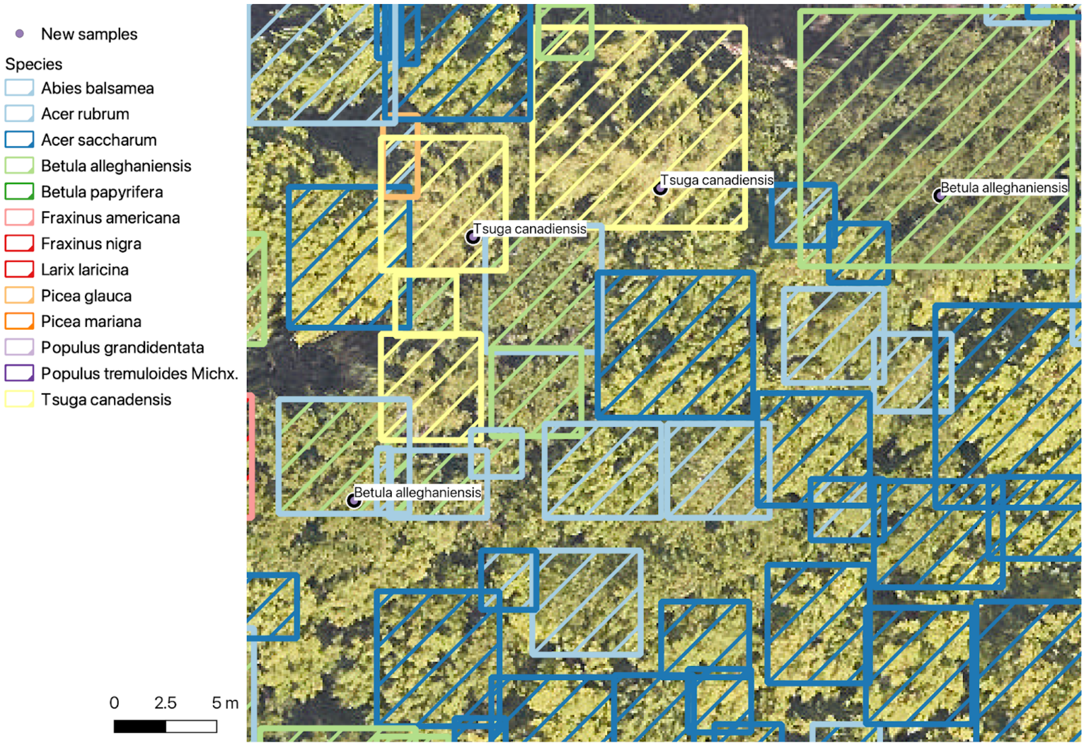

Box 1 Figure 1. Original versus re-trained model predictions for UNDE. Sample trees were collected in the field without guidance from the predictions. The outline color is the original label, the filled shade is the revised label. The two *Tsuga canadensis* (center) and the samples were correctly predicted in the original model. The *Betula allenghensis* samples were split, the tree on the right was correctly predicted in both models. The tree on the left was originally predicted as *Acer rubrum*, but was correctly predicted in the revised model. Overall, most labels do not change among models, with only a small number of trees changing labels. For example, several trees that were originally predicted as *Acer rubrum* have been revised, and a single *Picea glauca* was revised to *A. rubrum* in the top left.

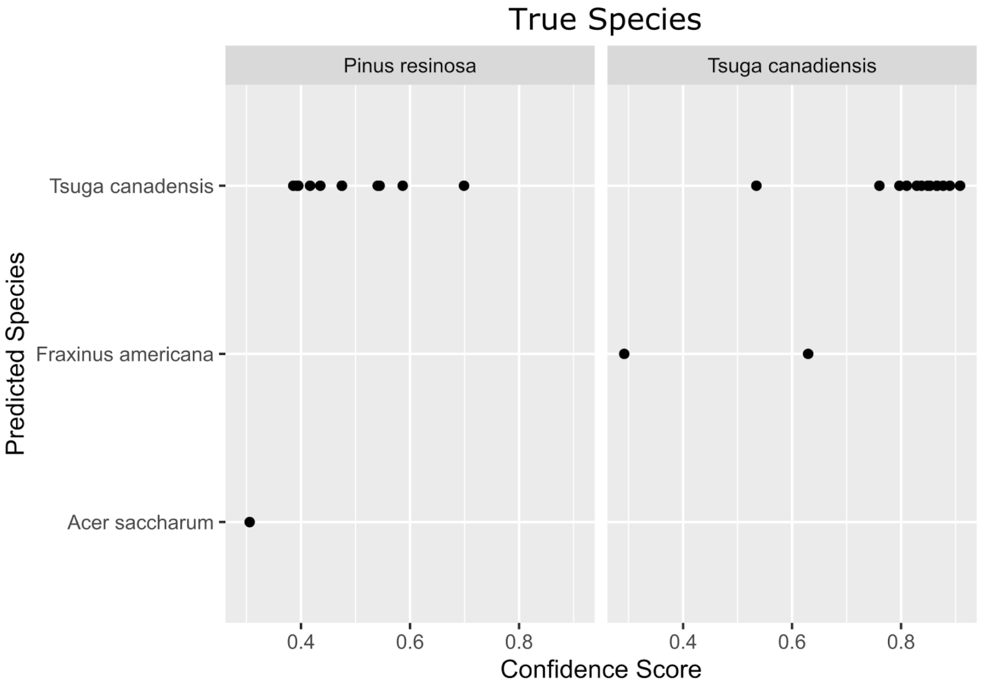

Box 1 Figure 2. Confidence scores for field-collected samples of *Pinus resinosa* and *Tsuga canadensis*. *P. resinosa* is a rare species at the site and we lacked sufficient samples to include it in the model. *T. canadiensis* is common throughout the site. The scores for the *P. resinosa* are lower than the score for *T. canadensis*, meaning that although we lack *P. resinosa* in the model, the features for the species may already exist within the model. This suggests that collecting additional data by targeting low confidence samples of common classes may be fruitful in uncovering existing diversity at the site.

How to make better models of tree species classification?

Through the process of generating species prediction for tens of millions of trees, we have experimented with many permutations of the model architecture and data workflow. As described above, the most pressing problem is data availability. Several days to weeks of targeted fieldwork often lead to improvements in species classification that cannot be achieved solely through changes to model or workflow. Secondly, the dominant source of uncertainty is in associating the tree stem with crown pixels. In general, models are superior in areas of open

forests with low diversity. The spacing among trees improves crown delineation and reduces the chance of any neighboring tree species polluting the spectral signature. This can be partially overcome in the fieldwork stage using crown polygons drawn on a tablet in the field, rather than solely using stem points taken by a GPS (Graves et al. 2022). The spectral pollution challenge occurs in both training and validation data, making it difficult to adopt many ‘weak labeling’ approaches common in computer vision literature that assume access to a small amount of confident examples and a larger set of less confidence samples. Development of unsupervised approaches to identify putative incorrect labels may be fruitful in rejecting incorrect matching between stem data and crown pixels. For this reason, many prior publications have focused solely on well spaced emergent tree crowns (e.g. Fricker et al. 2019). This may be useful in assessing algorithm performance, but risks overconfident depictions of algorithm performance that cannot be replicated when creating wall-to-wall landscape maps. Finally, we have found, given our approach, that the majority of classification performance is derived from the spectral signature of the crowns and not in the spatial structure of pixels in the crowns. Despite multiple publications that highlight performance gains through multi-modal data fusion, we did not find significant improvements when adding the 10cm RGB data.

Computer Vision uses of the dataset

Compared to the typical computer vision application, the data sample sizes of the classes used in these models are extremely low. The emerging area of research on ‘few shot learning’, in which foundation models are asked to predict new classes with only 1 to 5 samples, may be an avenue to conceive of tree species prediction rather than typical supervision used here (e.g. Sohn et al. 2020). In the extreme, the task of zero-shot learning, or unknown class detection, in which the model can identify classes not included in training, will have great utility in rapid biological surveys of new areas using models trained on NEON tree data.

Combining this challenge with the geographic extent of the study, there are several remaining computer vision challenges, including how to best create a single foundation model that is portable across space and time. Our approach used data from other sites to pretrain each site-specific model, but only included classes present at each target site. Marconi et al. (2022) used a portion of the training data to compare local versus global models for each site. Because of the differences in data, and evaluation approaches, a precise comparison between Marconi et al. (2022) and this article is not possible. We stress that the focus of this article is on the publication of the crowns dataset rather than a comparison of a bounding box multi-temporal deep learning approach versus the pixel-based ensemble of machine learning classifiers presented in Marconi et al. (2022).

There is considerable interest in developing species predictions for large areas using high resolution satellite and low cost UAV sensors. Most current satellites have coarser spectral resolution and the UAV sensors will have fewer, broader, spectral bands than the sensor used in this study. Using NEON data as a source for training data to project into these coarser resolution data has large benefits since the NEON data is both high spectral and spatial resolution. This kind of generalization study is often referred to as ‘Domain adaptation’ and is an open challenge in computer vision, with many proposed approaches to try to align either the input data or learned features among disparate sensors or geographic areas (Koh et al. 2021). Most approaches focus on a combination of supervised finetuning with unsupervised, or self-supervised learning, on the target domain. The ample unlabeled airborne data at NEON opens the possibility of a combination of supervised and unsupervised learning to increase transferability among geographic sites, spectral resolutions and spatial scales. The upload of the data to Google Earth Engine can facilitate very large scale data overlap among remote sensing assets on the Earth Engine catalog.

Acknowledgements

We would like to thank NEON staff and in particular Tristan Goulden and Courtney Meier for their assistance and support. We thank Natalie Heaton, Nicollete Lyons, Matthew Raulerson, Alex Seeley, Camille Sicangco, Luis Tirado and Stuart Wilkin and for field data collection efforts. This research was supported by the Gordon and Betty Moore Foundation's Data-Driven Discovery Initiative (GBMF4563) to EP White, by the USDA National Institute of Food and Agriculture McIntire Stennis project 1024612 and the Forest Systems Jumpstart program administered by the Florida Agricultural Experiment Station to SA Bohlman, and by the National Science Foundation (1926542) to EP White, SA Bohlman, A Zare, DZ Wang, and A Singh. This work was supported by the USDA National Institute of Food and Agriculture, Hatch project FLA-WEC-005944. PT acknowledges funding support from NSF Macrosystems Biology and NEON-Enabled Science (MSB-NES) award DEB 1638720 and NSF ASCEND Biology Integration Institute (BII) through DBI award 2021898. SR acknowledges funding support from NASA award 80NSSC23K0421 P00001 and Hatch project number ME022425. NGS and VER were supported by funding from NASA (80NSSC22K1625) and NSF Dimensions of Biodiversity (DEB-2124466). RAA was supported by the NWT LTER (NSF DEB-2224439), USDA NIFA McIntire Stennis project (1019284), and USDA NIFA postdoctoral award (2022-67012-37200).

Data Availability

The predictions, training data crops and shapefiles with predicted training crowns are available at https://zenodo.org/record/10067302. A csv file per site was uploaded to Google Earth engine and a public link is available as a FeatureCollection. For example, 'https://code.earthengine.google.com/?asset=users/benweinstein2010/RMNP' is the RMNP, Rocky Mountain National Park, predictions. For more on using NEON data and earth engine,

see https://www.neonscience.org/resources/learning-hub/tutorials/intro-aop-gee-image-collections.

References

Barnett, D. T., P. A. Duffy, D. S. Schimel, R. E. Krauss, K. M. Irvine, F. W. Davis, J. E. Gross, E. I. Azuaje, A. S. Thorpe, D. Gudex-Cross, M. Patterson, J. M. McKay, J. T. McCorkel, and C. L. Meier. 2019. The terrestrial organism and biogeochemistry spatial sampling design for the National Ecological Observatory Network. Ecosphere 10:e02540.

Davies, S. J., I. Abiem, K. Abu Salim, S. Aguilar, D. Allen, A. Alonso, K. Anderson-Teixeira, A. Andrade, G. Arellano, P. S. Ashton, P. J. Baker, M. E. Baker, J. L. Baltzer, Y. Basset, P. Bissiengou, S. Bohlman, N. A. Bourg, W. Y. Brockelman, S. Bunyavejchewin, D. F. R. P. Burslem, M. Cao, D. Cárdenas, L.-W. Chang, C.-H. Chang-Yang, K.-J. Chao, W.-C. Chao, H. Chapman, Y.-Y. Chen, R. A. Chisholm, C. Chu, G. Chuyong, K. Clay, L. S. Comita, R. Condit, S. Cordell, H. S. Dattaraja, A. A. de Oliveira, J. den Ouden, M. Detto, C. Dick, X. Du, Á. Duque, S. Ediriweera, E. C. Ellis, N. L. E. Obiang, S. Esufali, C. E. N. Ewango, E. S. Fernando, J. Filip, G. A. Fischer, R. Foster, T. Giambelluca, C. Giardina, G. S. Gilbert, E. Gonzalez-Akre, I. A. U. N. Gunatilleke, C. V. S. Gunatilleke, Z. Hao, B. C. H. Hau, F. He, H. Ni, R. W. Howe, S. P. Hubbell, A. Huth, F. Inman-Narahari, A. Itoh, D. Janík, P. A. Jansen, M. Jiang, D. J. Johnson, F. A. Jones, M. Kanzaki, D. Kenfack, S. Kiratiprayoon, K. Král, L. Krizel, S. Lao, A. J. Larson, Y. Li, X. Li, C. M. Litton, Y. Liu, S. Liu, S. K. Y. Lum, M. S. Luskin, J. A. Lutz, H. T. Luu, K. Ma, J.-R. Makana, Y. Malhi, A. Martin, C. McCarthy, S. M. McMahon, W. J. McShea, H. Memiaghe, X. Mi, D. Mitre, M. Mohamad, L. Monks, H. C. Muller-Landau, P. M. Musili, J. A. Myers, A. Nathalang, K. M. Ngo, N. Norden, V. Novotny, M. J. O'Brien, D. Orwig, R. Ostertag, K. Papathanassiou, G. G. Parker, R. Pérez, I. Perfecto, R. P. Phillips, N. Pongpattananurak, H. Pretzsch, H.

Ren, G. Reynolds, L. J. Rodriguez, S. E. Russo, L. Sack, W. Sang, J. Shue, A. Singh, G.-Z. M. Song, R. Sukumar, I.-F. Sun, H. S. Suresh, N. G. Swenson, S. Tan, S. C. Thomas, D. Thomas, J. Thompson, B. L. Turner, A. Uowolo, M. Uriarte, R. Valencia, J. Vandermeer, A. Vicentini, M. Visser, T. Vrska, X. Wang, X. Wang, G. D. Weiblen, T. J. S. Whitfeld, A. Wolf, S. J. Wright, H. Xu, T. L. Yao, S. L. Yap, W. Ye, M. Yu, M. Zhang, D. Zhu, L. Zhu, J. K. Zimmerman, and D. Zuleta. 2021. ForestGEO: Understanding forest diversity and dynamics through a global observatory network. Biological Conservation 253:108907.

Fricker, G. A., J. D. Ventura, J. A. Wolf, M. P. North, F. W. Davis, and J. Franklin. 2019. A Convolutional Neural Network Classifier Identifies Tree Species in Mixed-Conifer Forest from Hyperspectral Imagery. Remote Sensing 11:2326.

Hang, R., Z. Li, Q. Liu, P. Ghamisi, and S. S. Bhattacharyya. 2021. Hyperspectral Image Classification With Attention-Aided CNNs. IEEE Transactions on Geoscience and Remote Sensing 59:2281–2293.

Hastings, J. H., S. V. Ollinger, A. P. Ouimette, R. Sanders-DeMott, M. W. Palace, M. J. Ducey, F. B. Sullivan, D. Basler, and D. A. Orwig. 2020. Tree Species Traits Determine the Success of LiDAR-Based Crown Mapping in a Mixed Temperate Forest. Remote Sensing 12:309.

Koh, P. W., S. Sagawa, H. Marklund, S. M. Xie, M. Zhang, A. Balsubramani, W. Hu, M. Yasunaga, R. L. Phillips, I. Gao, T. Lee, E. David, I. Stavness, W. Guo, B. Earnshaw, I. Haque, S. M. Beery, J. Leskovec, A. Kundaje, E. Pierson, S. Levine, C. Finn, and P. Liang. 2021. WILDS: A Benchmark of in-the-Wild Distribution Shifts. Pages 5637–5664 Proceedings of the 38th International Conference on Machine Learning. PMLR.

Liu, Z., Z. Miao, X. Zhan, J. Wang, B. Gong, and S. X. Yu. 2019. Large-Scale Long-Tailed Recognition in an Open World. Pages 2532–2541 2019 IEEE/CVF Conference on Computer Vision and Pattern Recognition (CVPR). IEEE, Long Beach, CA, USA.

Marconi, S., B. G. Weinstein, S. Zou, S. A. Bohlman, A. Zare, A. Singh, D. Stewart, I. Harmon, A. Steinkraus, and E. P. White. 2022. Continental-scale hyperspectral tree species classification in the United States National Ecological Observatory Network. Remote Sensing of Environment 282:113264.

Maschler, J., C. Atzberger, and M. Immitzer. 2018. Individual Tree Crown Segmentation and Classification of 13 Tree Species Using Airborne Hyperspectral Data. Remote Sensing 10:1218.

National Ecological Observatory Network (NEON). 2021. Woody plant vegetation structure (DP1.10098.001). National Ecological Observatory Network (NEON).

Schäfer, E., J. Heiskanen, V. Heikinheimo, and P. Pellikka. 2016. Mapping tree species diversity of a tropical montane forest by unsupervised clustering of airborne imaging spectroscopy data. Ecological Indicators 64:49–58.

Sohn, K., D. Berthelot, N. Carlini, Z. Zhang, H. Zhang, C. A. Raffel, E. D. Cubuk, A. Kurakin, and C.-L. Li. 2020. FixMatch: Simplifying Semi-Supervised Learning with Consistency and Confidence. Pages 596–608 Advances in Neural Information Processing Systems. Curran Associates, Inc.

Tucker, C., M. Brandt, P. Hiernaux, A. Kariryaa, K. Rasmussen, J. Small, C. Igel, F. Reiner, K. Melocik, J. Meyer, S. Sinno, E. Romero, E. Glennie, Y. Fitts, A. Morin, J. Pinzon, D. McClain, P. Morin, C. Porter, S. Loeffler, L. Kergoat, B.-A. Issoufou, P. Savadogo, J.-P. Wigneron, B. Poulter, P. Ciais, R. Kaufmann, R. Myneni, S. Saatchi, and R. Fensholt. 2023. Sub-continental-scale carbon stocks of individual trees in African drylands. Nature 615:80–86.

Veblen, T., R. Andrus, and R. Chai. 2021. Permanent forest plot data from 1982-2019 at Niwot Ridge. Environmental Data Initiative.

Wallis, C. I. B., A. L. Crofts, D. Inamdar, J. P. Arroyo-Mora, M. Kalacska, É. Laliberté, and M. Vellend. 2023. Remotely sensed carbon content: The role of tree composition and tree

diversity. Remote Sensing of Environment 284:113333.

Weinstein, B. G., S. Marconi, M. Aubry-Kientz, G. Vincent, H. Senyondo, and E. P. White. 2020a. DeepForest: A Python package for RGB deep learning tree crown delineation. Methods in Ecology and Evolution 11:1743–1751.

Weinstein, B. G., S. Marconi, S. A. Bohlman, A. Zare, and E. P. White. 2020b. Cross-site learning in deep learning RGB tree crown detection. Ecological Informatics 56:101061.

Weinstein, B. G., S. Marconi, S. J. Graves, A. Zare, A. Singh, S. A. Bohlman, L. Magee, D. J. Johnson, P. A. Townsend, and E. P. White. 2023. Capturing long-tailed individual tree diversity using an airborne imaging and a multi-temporal hierarchical model. Remote Sensing in Ecology and Conservation n/a.

Table S1: Species included in each model for each NEON site. The number of samples (n) for each species in the canopy filtered data. To be included in the model, a species needs to have atleast 10 training samples and 10 test samples at a site in the final filtered data. The number of predicted trees at each site, the proportion of total predictions at the site, and the rank abundance of each species is shown.

sci_name	count	site	proportion	rank	Habitat
Tsuga canadensis (L.) Carrire	1916656	BART	0.44	1	Northern Broadleaf
Fagus grandifolia Ehrh.	557790	BART	0.13	2	Northern Broadleaf
Acer saccharum Marshall	529581	BART	0.12	3	Northern Broadleaf
Betula alleghaniensis Britton	527648	BART	0.12	4	Northern Broadleaf
Acer rubrum L.	384249	BART	0.09	5	Northern Broadleaf
Fraxinus americana L.	317331	BART	0.07	6	Northern Broadleaf
Betula papyrifera Marshall	119675	BART	0.03	7	Northern Broadleaf
Juglans nigra L.	856379	BLAN	0.35	1	Southern Broadleaf
Cornus florida L.	435672	BLAN	0.18	2	Southern Broadleaf
Celtis occidentalis L.	348581	BLAN	0.14	3	Southern Broadleaf
Quercus rubra L.	260101	BLAN	0.11	4	Southern Broadleaf
Liriodendron tulipifera L.	217721	BLAN	0.09	5	Southern Broadleaf
Pinus strobus L.	202535	BLAN	0.08	6	Southern Broadleaf
Platanus occidentalis L.	92087	BLAN	0.04	7	Southern Broadleaf
Picea mariana (Mill.) Britton, Sterns & Poggenb.	3858957	BONA	0.51	1	Conifer
Betula neoalaskana Sarg.	2365676	BONA	0.31	2	Conifer
Populus tremuloides Michx.	923889	BONA	0.12	3	Conifer
Picea glauca (Moench) Voss	424178	BONA	0.06	4	Conifer
Quercus stellata Wangenh.	1276070	CLBJ	0.60	1	Savannah

Juniperus virginiana L.	488138	CLBJ	0.23	2	Savannah
Quercus marilandica M√ºnchh.	375595	CLBJ	0.18	3	Savannah
Picea glauca (Moench) Voss	1282877	DEJU	0.37	1	Conifer
Picea mariana (Mill.) Britton, Sterns & Poggenb.	1223419	DEJU	0.36	2	Conifer
Populus tremuloides Michx.	920182	DEJU	0.27	3	Conifer
Pinus taeda L.	786987	DELA	0.22	1	Southern Broadleaf
Quercus nigra L.	756838	DELA	0.21	2	Southern Broadleaf
Acer rubrum L.	675465	DELA	0.19	3	Southern Broadleaf
Liquidambar styraciflua L.	478992	DELA	0.13	4	Southern Broadleaf
Fraxinus pennsylvanica Marshall	370191	DELA	0.10	5	Southern Broadleaf
Celtis laevigata Willd.	289826	DELA	0.08	6	Southern Broadleaf
Carya tomentosa (Lam.) Nutt.	262398	DELA	0.07	7	Southern Broadleaf
Acer rubrum L.	1142770	GRSM	0.47	1	Southern Broadleaf
Quercus montana Willd.	851493	GRSM	0.35	2	Southern Broadleaf
Liriodendron tulipifera L.	453621	GRSM	0.19	3	Southern Broadleaf
Acer rubrum L.	2092750	HARV	0.23	1	Northern Broadleaf
Pinus strobus L.	1928207	HARV	0.21	2	Northern Broadleaf
Quercus rubra L.	1656996	HARV	0.18	3	Northern Broadleaf
Quercus alba L.	1192530	HARV	0.13	4	Northern Broadleaf
Betula lenta L.	743261	HARV	0.08	5	Northern Broadleaf
Fraxinus americana L.	561297	HARV	0.06	6	Northern Broadleaf
Tsuga canadensis (L.) Carri√®re	419849	HARV	0.05	7	Northern Broadleaf
Betula alleghaniensis Britton	257040	HARV	0.03	8	Northern Broadleaf
Pinus resinosa Aiton	91640	HARV	0.01	9	Northern Broadleaf
Fagus grandifolia Ehrh.	68370	HARV	0.01	10	Northern Broadleaf

Prunus serotina Ehrh.	55833	HARV	0.01	11	Northern Broadleaf
Picea abies (L.) Karst.	27501	HARV	0.00	12	Northern Broadleaf
Nyssa sylvatica Marshall	26159	HARV	0.00	13	Northern Broadleaf
Quercus velutina Lam.	1259	HARV	0.00	14	Northern Broadleaf
Pinus palustris Mill.	2159896	JERC	0.58	1	Savannah
Quercus hemisphaerica W. Bartram ex Willd.	1280439	JERC	0.35	2	Savannah
Quercus margaretta	257687	JERC	0.07	3	Savannah
Liquidambar styraciflua L.	2153931	LENO	0.63	1	Southern Broadleaf
Quercus pagoda Raf.	886055	LENO	0.26	2	Southern Broadleaf
Quercus nigra L.	396110	LENO	0.12	3	Southern Broadleaf
Quercus rubra L.	1610301	MLBS	0.43	1	Southern Broadleaf
Acer rubrum L.	873498	MLBS	0.23	2	Southern Broadleaf
Quercus alba L.	475352	MLBS	0.13	3	Southern Broadleaf
Liriodendron tulipifera L.	411167	MLBS	0.11	4	Southern Broadleaf
Quercus coccinea M√ºnchh.	359546	MLBS	0.10	5	Southern Broadleaf
Pinus contorta Douglas ex Loudon	2005920	NIWO	0.43	1	Conifer
Picea engelmannii Parry ex Engelm.	1268215	NIWO	0.27	2	Conifer
Abies lasiocarpa (Hook.) Nutt.	999052	NIWO	0.22	3	Conifer
Pinus flexilis James	354697	NIWO	0.08	4	Conifer
Quercus hemisphaerica W. Bartram ex Willd.	464452	OSBS	0.13	1	Savannah
Pinus palustris Mill.	459380	OSBS	0.13	2	Savannah
Pinus taeda L.	441837	OSBS	0.13	3	Savannah
Pinus elliottii Engelm.	428306	OSBS	0.12	4	Savannah
Quercus laevis Walter	315498	OSBS	0.09	5	Savannah

Quercus geminata Small	274916	OSBS	0.08	6	Savannah
Quercus virginiana Mill.	228768	OSBS	0.07	7	Savannah
Pinus clausa (Chapm. ex Engelm.) Vasey ex Sarg.	189431	OSBS	0.05	8	Savannah
Liquidambar styraciflua L.	162751	OSBS	0.05	9	Savannah
Nyssa sylvatica Marshall	145971	OSBS	0.04	10	Savannah
Magnolia sp.	132960	OSBS	0.04	11	Savannah
Quercus nigra L.	84128	OSBS	0.02	12	Savannah
Acer rubrum L.	73579	OSBS	0.02	13	Savannah
Carya glabra (Mill.) Sweet	61204	OSBS	0.02	14	Savannah
Pinus contorta Douglas ex Loudon	2656483	RMNP	0.35	1	Conifer
Pinus ponderosa Lawson & C. Lawson	1491041	RMNP	0.20	2	Conifer
Pseudotsuga menziesii (Mirb.) Franco	1185424	RMNP	0.16	3	Conifer
Pinus flexilis James	812500	RMNP	0.11	4	Conifer
Populus tremuloides Michx.	513952	RMNP	0.07	5	Conifer
Picea engelmannii Parry ex Engelm.	419298	RMNP	0.06	6	Conifer
Abies lasiocarpa (Hook.) Nutt.	408857	RMNP	0.05	7	Conifer
Liriodendron tulipifera L.	614569	SERC	0.27	1	Southern Broadleaf
Liquidambar styraciflua L.	503363	SERC	0.22	2	Southern Broadleaf
Acer rubrum L.	256490	SERC	0.11	3	Southern Broadleaf
Quercus falcata Michx.	236338	SERC	0.10	4	Southern Broadleaf
Fraxinus pennsylvanica Marshall	179711	SERC	0.08	5	Southern Broadleaf
Platanus occidentalis L.	130393	SERC	0.06	6	Southern Broadleaf
Quercus velutina Lam.	117590	SERC	0.05	7	Southern Broadleaf

Quercus alba L.	91438	SERC	0.04	8	Southern Broadleaf
Fagus grandifolia Ehrh.	87899	SERC	0.04	9	Southern Broadleaf
Carya tomentosa (Lam.) Nutt.	44351	SERC	0.02	10	Southern Broadleaf
Quercus wislizeni A. DC.	410332	SJER	0.48	1	Savannah
Quercus douglasii Hook. & Arn.	325524	SJER	0.38	2	Savannah
Pinus sabiniana Douglas ex Douglas	116312	SJER	0.14	3	Savannah
Calocedrus decurrens (Torr.) Florin	1957264	SOAP	0.65	1	Conifer
Pinus ponderosa Lawson & C. Lawson	626354	SOAP	0.21	2	Conifer
Quercus kelloggii Newberry	242541	SOAP	0.08	3	Conifer
Quercus chrysolepis Liebm.	198656	SOAP	0.07	4	Conifer
Abies balsamea (L.) Mill.	2390776	STEI	0.36	1	Northern Broadleaf
Populus tremuloides Michx.	1407230	STEI	0.21	2	Northern Broadleaf
Tilia americana L.	1017129	STEI	0.15	3	Northern Broadleaf
Acer rubrum L.	846965	STEI	0.13	4	Northern Broadleaf
Quercus rubra L.	721269	STEI	0.11	5	Northern Broadleaf
Acer saccharum Marshall	304397	STEI	0.05	6	Northern Broadleaf
Quercus alba L.	1128147	TALL	0.29	1	Southern Broadleaf
Pinus taeda L.	1007905	TALL	0.26	2	Southern Broadleaf
Pinus palustris Mill.	744862	TALL	0.19	3	Southern Broadleaf
Liquidambar styraciflua L.	554307	TALL	0.14	4	Southern Broadleaf
Pinus echinata Mill.	330868	TALL	0.09	5	Southern Broadleaf
Liriodendron tulipifera L.	122261	TALL	0.03	6	Southern Broadleaf
Pinus contorta Douglas ex	1238741	TEAK	0.34	1	Conifer

Loudon					
Abies concolor (Gord. & Glend.) Lindl. ex Hildebr.	766721	TEAK	0.21	2	Conifer
Pinus jeffreyi Balf.	520066	TEAK	0.14	3	Conifer
Abies lowiana (Gordon & Glend.) A. Murray bis	439012	TEAK	0.12	4	Conifer
Abies magnifica A. Murray bis	423231	TEAK	0.12	5	Conifer
Calocedrus decurrens (Torr.) Florin	155247	TEAK	0.04	6	Conifer
Pinus lambertiana Douglas	117623	TEAK	0.03	7	Conifer
Quercus rubra L.	1117038	TREE	0.16	1	Northern Broadleaf
Abies balsamea (L.) Mill.	1009185	TREE	0.14	2	Northern Broadleaf
Populus tremuloides Michx.	982204	TREE	0.14	3	Northern Broadleaf
Betula papyrifera Marshall	713772	TREE	0.10	4	Northern Broadleaf
Picea mariana (Mill.) Britton, Sterns & Poggenb.	572793	TREE	0.08	5	Northern Broadleaf
Acer saccharum Marshall	494667	TREE	0.07	6	Northern Broadleaf
Tilia americana L.	449669	TREE	0.06	7	Northern Broadleaf
Larix laricina (Du Roi) K. Koch	437108	TREE	0.06	8	Northern Broadleaf
Pinus resinosa Aiton	359730	TREE	0.05	9	Northern Broadleaf
Thuja occidentalis L.	288164	TREE	0.04	10	Northern Broadleaf
Picea glauca (Moench) Voss	206034	TREE	0.03	11	Northern Broadleaf
Acer rubrum L.	191871	TREE	0.03	12	Northern Broadleaf
Fraxinus pennsylvanica Marshall	125480	TREE	0.02	13	Northern Broadleaf
Pinus strobus L.	122127	TREE	0.02	14	Northern Broadleaf
Tsuga canadensis (L.) Carri√®re	48679	TREE	0.01	15	Northern Broadleaf
Maclura pomifera (Raf.) C.K. Schneid.	399538	UKFS	0.25	1	Southern Broadleaf

Juniperus virginiana L.	352218	UKFS	0.22	2	Southern Broadleaf
Celtis occidentalis L.	301882	UKFS	0.19	3	Southern Broadleaf
Ulmus americana L.	163371	UKFS	0.10	4	Southern Broadleaf
Quercus muehlenbergii Engelm.	143089	UKFS	0.09	5	Southern Broadleaf
Carya ovata (Mill.) K. Koch	91871	UKFS	0.06	6	Southern Broadleaf
Gleditsia triacanthos L.	82320	UKFS	0.05	7	Southern Broadleaf
Juglans nigra L.	68406	UKFS	0.04	8	Southern Broadleaf
Acer saccharum Marshall	727435	UNDE	0.15	1	Northern Broadleaf
Picea mariana (Mill.) Britton, Sterns & Poggenb.	686836	UNDE	0.14	2	Northern Broadleaf
Picea glauca (Moench) Voss	528496	UNDE	0.11	3	Northern Broadleaf
Tsuga canadensis (L.) Carri√®re	476682	UNDE	0.10	4	Northern Broadleaf
Abies balsamea (L.) Mill.	432414	UNDE	0.09	5	Northern Broadleaf
Betula alleghaniensis Britton	432101	UNDE	0.09	6	Northern Broadleaf
Acer rubrum L.	399475	UNDE	0.08	7	Northern Broadleaf
Betula papyrifera Marshall	323397	UNDE	0.07	8	Northern Broadleaf
Larix laricina (Du Roi) K. Koch	270436	UNDE	0.06	9	Northern Broadleaf
Populus tremuloides Michx.	168410	UNDE	0.03	10	Northern Broadleaf
Populus grandidentata Michx.	155654	UNDE	0.03	11	Northern Broadleaf
Fraxinus nigra Marshall	130453	UNDE	0.03	12	Northern Broadleaf
Fraxinus americana L.	89307	UNDE	0.02	13	Northern Broadleaf
Pseudotsuga menziesii (Mirb.) Franco	3987937	WREF	0.59	1	Conifer
Tsuga heterophylla (Raf.) Sarg.	1218052	WREF	0.18	2	Conifer
Abies amabilis (Douglas ex Loudon) Douglas ex Forbes	903627	WREF	0.13	3	Conifer
Thuja plicata Donn ex D. Don	664059	WREF	0.10	4	Conifer

Pinus contorta Douglas ex Loudon	3326783	YELL	0.74	1	Conifer
Pseudotsuga menziesii (Mirb.) Franco	889082	YELL	0.20	2	Conifer
Populus tremuloides Michx.	262641	YELL	0.06	3	Conifer
