# Supplementary material for: Individual canopy tree species maps for the National Ecological Observatory Network
Source: PLoS Biol. 2024 Jul 16;22(7):e3002700. doi: 10.1371/journal.pbio.3002700 (PMC11251727; doi:10.1371/journal.pbio.3002700)
Supplement: Attachment — Submitted filename: PLOS Biology Reviews.pdf [file pbio.3002700.s008.pdf]

Dr. Roberts,

Thank you for the opportunity to improve this manuscript. We have restructured the Introduction, Methods, and Discussion to be much shorter and clearer while covering the current state of the field in more detail. We have added the new analyses requested by Reviewer #2 and Reviewer #3 to quantify the robustness and limitations of the approach. We have also made changes to the language throughout the manuscript to highlight the appropriate use and the limitations of the resulting data to address Reviewer #3's concerns. Specifically, we have made the following revisions:

- **Shortened the Introduction and focused on highlighting key innovations in tree detection and species classifications. We have also added a brief summary of the technical articles in our previous work to provide context for the published evaluation performance of the different components.**
- **Shortened the Discussion to highlight our key findings about the patterns of model performance and outline next steps for model improvement. We have also added an explicit paragraph highlighting the limitations of the dataset and how it should be used.**
- **Expanded the citations for highlighting previous work, as well as greater support for the credibility of the evaluation pipeline.**
- **Added several supplementary analyses including: 1) an evaluation of RGB + HSI integration experiments that shows a lack of improvement from adding RGB data to tree species classification; 2) an evaluation of the choice of single-site models compared to a single model for all sites, which shows that this decision resulted in more accurate predictions; and 3) comparisons of our canopy tree predictions to tree counts that include non-canopy trees to remind downstreams users that this data is only for canopy trees. These additional analyses all validate the modeling choices originally presented in the manuscript and we appreciate the opportunity to justify these decisions more clearly.**
- **Provided links to the already publicly available open source code**
- **We have completed a web visualization to allow users to view the data. https://visualize.idtrees.org/**

We have responded to the reviewer comments below. Given the extensive text revisions, there are many editorial comments made by reviewers that pertain to text that has since been removed. We have added a track changes document for review as well.

Sincerely,

Ben Weinstein, Sergio Marconi, Alina Zare, Stephanie Bohlman, Aditya Singh, Sarah Graves, Lukas Magee, Daniel Johnson, Sydne Record, Vanessa Rubio, Nathan Swenson, Philip Townsend, Thomas Veblen, Robert Andrus, and Ethan P. White

Dear Dr Weinstein,

Thank you for your patience while your manuscript "Individual tree species maps for the National Ecological Observatory Network" was peer-reviewed at PLOS Biology. It has now been evaluated by the PLOS Biology editors, an Academic Editor with relevant expertise, and by three independent reviewers.

You'll see that reviewer #1 says that your study is important and “of great merit,” but wants re-structuring of the Intro (including better coverage of the existing literature) and some clarification of the methodology. Reviewer #2 is also positive, but wonders if your could test the robustness of several aspects of your approach. Reviewer #3 is more critical; s/he appreciates the scale but is less impressed by the quality, urging you to flag the limitations more prominently so that readers aren’t misled into thinking that the dataset is better than it is. As part of this, s/he wants them to quantify the biases inherent in their dataset. Like reviewer #1, reviewer #3 thinks that much of the Intro belongs in the M&M, and that instead the Intro should cover the current state of the field in more detail; the Discussion should contain an account of the limitations. And you also need to provide the code (we would insist on this anyway).

In light of the reviews, which you will find at the end of this email, we would like to invite you to revise the work to thoroughly address the reviewers' reports.

Given the extent of revision needed, we cannot make a decision about publication until we have seen the revised manuscript and your response to the reviewers' comments. Your revised manuscript is likely to be sent for further evaluation by all or a subset of the reviewers.

Reviewer #1:

The paper describes a database of identified and species-classified tree crowns across 24 National Ecology Observation Network (NEON) sites in the US. The distribution of tree species and how it relates to both climatic, geological, and other environmental variables is an important area of study. The database, if made available publicly, would be of great merit to the ecological community. The manuscript is well-written and reads well but would benefit from a restructuring of the introduction and some clarification over several steps in the methods. I would recommend publication after some corrections.

Major issues

The current four paragraphs of the introduction are structured as follows:

(1) Individual tree crown (ITC) based dataset on tree species distribution is valuable in ecology, forest management, and global change biology
(2) NEON provides a high-resolution remote-sensing dataset covering 10000s of hectares of forests
(3) The NEON dataset was used to identify 100 million trees using a modified method from Weinstein et al. (2023)
(4) The structure of the dataset and how the methods improved on existing deep learning workflows
Paragraphs (2), (3), and (4) contain a lot of information on how this study was conducted, which should largely be moved to the methods section. On the other hand, the introduction has yet to provide a proper account of existing work in the field. There is a large body of research that attempted individual tree crown species classification using different types of remote sensing data. For instance, Fassnacht et al. (2016) reviewed >100 studies that used hyperspectral, multispectral, LiDAR, or a combination of RS datasets to classify trees into species. The authors claimed that the existing studies have not applied the models in large geographical areas across biomes (L90-94) and that Weinstein et al. (2023) provides a better workflow to handle datasets covering wide geographical areas (L94-108). While this may well be the case, the paragraph includes no citations to previous studies, which is needed before highlighting the research gap. Overall, the introduction would benefit from restructuring and the addition of proper citations.

We have significantly revised the Introduction to make it shorter, move details to the methods section, and highlight important previous work surrounding the key innovations in tree detection (L64) and tree species classification (L54) as well as the key previous work from our group that directly relates to model performance and evaluation of the tree maps (L176).

Minor issues

1. L55-57: This may be obvious to the authors, but adding citations to previous studies that did this would be nice. L57-60: Please cite some examples here.

This was an oversight on our part. We have significantly expanded the citations in the revised Introduction to highlight recent examples of large scale forest research using individual trees predicted from airborne data.

2. L119: Is "RGB camera mosaic" the correct terminology here? Is this the same as "mosaiced RGB imagery"? The term appeared several times after as well.

“RGB camera mosaic” comes from the NEON documentation of the data product, but we agree that this technical jargon is unnecessary and have replaced it with ‘RGB imagery’, which is simpler and sufficiently clear. The one exception is on line L111 when we directly reference NEON’s data.

3. L121: What does it mean by 70% accuracy between predicted and hand-annotated tree crowns? Does that mean 70% of the crowns have intersection over union scores over 0.5? Not a must to provide a full explanation to how this figure was derived, but if it could be succinctly summarized in a sentence or two it would be useful to include as this could quite heavily affect dataset usability.

We have added a paragraph outlining the previous work with greater attention to explaining previous evaluation experiments. L176

4. L188-189: Missing comma. Also consider adding a sentence before this to make it easier to understand. Something along the lines of "Many species captured in the original dataset would be lost after data filtering". L188-198: Overall, this paragraph is difficult to follow. At the start of the paragraph, the authors were referring to the filtered tree stem dataset collected from the field. It then jumped to "species captured by the model". While I understand that the species in the filtered dataset are the ones included in the species classification models, up to this point, the model has not been mentioned or described yet, which makes it very confusing. I would suggest either (1) making it clear at the very start that you are using the species to build a model, and that you want to evaluate the proportion of species the model captures, or, alternatively, (2) describing the filtered tree stems as "training data" and don't refer to the model until the next paragraph. On a more positive note, the hypothetical example at the end of the paragraph (L195-198) was a nice touch, made things much clearer.

We have rewritten this section (L159) to reduce text and be more direct about the tradeoff between ensuring high quality training data while keeping as many species in a model as possible. We have also updated Figure 3 to include both ‘all trees’ and ‘canopy trees’ included in the model.

5. L203-205: What does it mean by optionally classifying trees into "Broadleaf", "Conifer" and "Dominant species class"? Dominant species would either be broadleaved or coniferous. Did you have one model per site? If so, this should be stated at the very start.

We built one model per NEON site (L210). The most common species in the data is considered the dominant class if it consists of more than 40% of the training individuals (L223), regardless of whether it is a broadleaf or conifer. We have significantly shortened the methods to not distract the reader who might miss these key points.

6. L219-223: Were multiple models built using different hyperspectral datasets? A big issue in hyperspectral remote sensing is that models are often not very transferrable across datasets, especially if the imagery was collected in different seasons when the spectral signature of trees would be complicated by phenology.

We have addressed this question by clarifying up front that each site has its own set of hierarchical models (L210). NEON collects airborne data at approximately the same time each year to minimize phenological differences and phenological variation is built into the model via the multi-year ensemble. We agree that this can limit transferability to different locations or seasons,

but neither of those is a goal of this initial application. We have provided expanded justification for the decision to model sites separately based on improved prediction and therefore a better dataset, which is the goal of this paper, in a new supplementary analysis, see the new figure S2, as well as responses to Reviewer 3's questions.

7. L223-225: Needs a citation

We have added more citations throughout. This particular line was removed in revision.

8. L245: Is a 40m buffer enough to prevent spatial autocorrelation from affecting accuracy measurements?

We believe 40m is enough to ensure there are no overlapping crowns among contributed data areas. It is also the size of the NEON terrestrial sampling plots. Given the field sampling design implemented by NEON, using a larger buffer would reduce the number of species that could be included in the models, so we think this number is a reasonable compromise.

9. L273: 3.56 million trees per site across 24 sites don't give 100,021,471 trees. Or were there trees outside the sites?

We appreciate the reviewer noticing this, we incorrectly calculated the number of trees based on the csv files. We have regenerated this data and recalculated the total number of trees, it is 103,441,970 with an average of 4.31 million, updated on L288. The sites vary widely in both area and density, with sites like San Joaquin Valley, CA (SJER), having both sparse density and less area (0.8 million trees), Bartlett Experiment Forest, New Hampshire (BART) with average density (4.3 million trees) and very large sites like TreeHaven, Wisconsin (TREE) that cover large areas (7.1 million trees). We have added text to highlight this large range in tree counts.

10. L352-354: Citations needed

We have added citations on recent model developments in individual tree detection and classification L56, as well as a citation of recent work on full end-to-end workflows (L62, L190).

11. L356-357:

This section was removed during revision.

12. L356-369: The authors made a good point about current studies often being overly focused on accuracy figures over small and neat datasets. I agree that a model that produces reasonable estimates with 70% OA for large areas would be more useful than one that gets 99% in Indiana Pines. It is also true that many studies are trained and validated with manually segmented crowns, which generate accuracy figures that do not account for errors in automatic tree segmentation. However, I have some reservations regarding the authors' claim that the accuracy figures presented here are better at accounting for these errors. The ~30% error associated with generating the DeepForest bounding boxes is arguably not incorporated into the accuracy figures presented in this study. If perfect manually delineated and species-identified crows were used as validation, the classification errors would be substantially higher. I don't think a dataset needs to be perfect to be useful, and the authors might have done the best they could with the dataset

available, but it might be a stretch to claim that the accuracy figures presented here are more representative than those in previous work.

We appreciate this feedback, and we agree with the reviewer's view that, 1) there is value in dealing with the messiness of real ecological data, 2) there is a genuine reason why previous work have used stem data to anchor evaluation, since it does help disentangle the competing factors, 3) the approach we've adopted doesn't free us from bias, it just adds a different kind of bias, since DeepForest boxes are of course not perfect.

The general revision of the discussion was not to focus on fine technical details, but provide several paragraphs outlining the uncertainty in the workflow, and cite examples of how we can model and use these data (e.g. L408).

Reviewer #2:

The authors present an interesting study on using deep learning models to create species predictions for individual trees for 24 sites in the National Ecological Observatory Network. Overall I think it is a fine manuscript and I do not have major criticisms.

I'm happy to see that the output is shared publicly via Google Earth Engine too.

- A potential downside of combining high resolution imagery data, lidar, and hyperspectral data is that not all of these are available outside the study area, which makes it hard to extend to other regions, and as a result the transferability of the model is difficult. I understand that including the best available data to make the best possible model was the main aim of this project, but it might be interesting to compare the outcome of a model that has, for example, only RGB imagery included. A recent paper in remote sensing (https://www.mdpi.com/2072-4292/15/5/1463) did exactly this and had reasonable accuracy statistics as far as I can tell.

The focus of this paper is to provide researchers at NEON the best possible data. Our decisions regarding modeling approach emphasize predictive accuracy over transferability. Comparing models based on different underlying data is not yet the focus of our modeling approach. We have made this clearer in text, L211, L486, Figure S2. We are certainly interested in model transferability outside of NEON and are pursuing this in other work. We have also added a figure and text in the supplementary materials L783, Figure S1, to show that initial attempts at RGB models were not successful.

A similar point for creating a single model per site. I think this is a valid decision, but it would be interesting to see how much worse (if at all?) a single model would perform.

We have added a new analysis showing that a single model results in less accurate predictions (Figure S2) and explained that we chose this approach to provide the most accurate predictions possible. See the revised Discussion and L486.

- The number of training samples for some of the sites, (e.g. SJER, LENO, CLBJ) is very small. Is it realistic to train a model on so few samples?

We agree with the reviewer that several sites have low sample sizes relative to standard deep learning applications. We have revised the discussion to clearly underline the low sample sizes

(e.g. Box 1, L427). Due to this limitation in the NEON data there is a tradeoff between producing reasonable predictions and including as many sites as possible. We have updated Figure 3 to show the proportion of all tree species, versus those judged to be present in the canopy. Based on our evaluation of this tradeoff, we removed sites like Jones River Environmental Center (JERC) due to too few samples, but kept several sites with slightly more training data. The next lowest sampled site after JERC, San Joaquin Experimental Range (SJER), has very low species diversity but the models perform well, so we drew the line there and provided information on data sizes and accuracy metrics so that others could decide whether to use the resulting data product for each site given their use case.

To make the limitations to the predicted data associated with this and other issues more transparent to the ready, we have extensively revised the manuscript to remind users about the limitations of the dataset (paragraphs L375, L386, L408). For example, just because SJER has 100% test accuracy, does not mean we anticipate that every one of the 850,000 trees at the site is correctly identified. We have added discussion to try to be transparent about the current state of the models and resulting predictions. We hope that including sites with lower sample sizes will help serve as a call to the community to contribute additional field data allowing us to improve and update these predictions following the approaches highlighted in Box 1.

- General comment- the site names are mentioned throughout the manuscript but for people not familiar with NEON

We have replaced NEON site codes with full names whenever space allows, especially in Figure legends.

Reviewer #3:
[IMPORTANT: please note this reviewers' additional comments in the attached PDF]

In the manuscript entitled "Individual tree species maps for the National Ecological Observatory Network", the authors introduce a new dataset generated using a rather large dataset using a rather complex methodology. While I think that any effort towards creating open and ML-ready datasets represents a great step in science I have some major concerns regarding this study that I think should be addressed by the authors prior to publication.

Generally, it seems that the authors aim at generating a very large dataset rather than creating a well-curated dataset. From personal experience and from literature is evident that, if on one hand deep learning models can leverage large datasets, the quality of the labels remains the most important aspect to train models that are reliable and hence do not inherit inherent biases and quality issues caused by having poor training data. This aspect, which due to inherent under detection of trees in RGB images (cannot detect and classify species for understory and partly dominated trees) is clearly an issue in the dataset presented in this paper, and can cause serious problems in downstream applications. In particular, it is rather dangerous when the users are not well aware of the limitations of the dataset. Hence, I urge the authors to clearly highlight the limitation of the proposed dataset, which is composed of pseudo-labelled trees (i.e. predicted from a deep learning
models and not manually annotated), artificially generated trees (i.e. trees with 1 m buffer bounding box created according to lines 183-184). This would be best clarified by including a separate subsection in the discussion section to highlight the dataset key limitations.

We agree with the reviewer that it is important to clearly lay out limitations to the dataset and its use, so that downstream users can properly evaluate whether the resulting data is appropriate for their applications. We have extensively revised the manuscript to discuss the limitations of the dataset, and point to citations that will be useful in capturing the uncertainty in each stage of the workflow (paragraphs L375, L386, L408).

As the reviewer notes, the use of airborne remote sensing inherently limits the data to canopy trees, a common limitation in all airborne forest remote sensing. We have revised Figure 3 to include 'all trees', and then those just just judged to be within the canopy.

In terms of general tone, the revision focused on being clearer about 'canopy tree prediction', e..g L27, L92, and updating the title. Beyond this limitation we also agree that not all 100 million predictions will be accurate, but believe having initial maps is critical to forming hypotheses, motivating additional sampling, and developing the downstream tools to handle model uncertainty. We have added the suggested paragraph to the Discussion, as well as done a better job of highlighting the work in prior manuscripts addressing uncertainty in tree detection.

We note that while there are potential downstream deep learning applications for this dataset (e.g., upscaling to satellite imagery), its primary value is the direct use of the associated predictions for understanding ecosystems at scales that are not possible using field data alone. We reduced any text that felt as if field-data and airborne data are mutually exclusive or competing in some way. We see airborne data as a complement to field surveys (L375).

Here is critical that the authors report and quantify the:

a) under-detection of trees using the bounding box model in terms of detection rate (or estimation of tree numbers) compared to field data.

We have previously quantified the detection rates of ~70% of canopy trees based on field identified stems in Weinstein et al. 2021 and have now referenced this in the manuscript (L187). Our understanding is that the reviewer wants to see a comparison of counts to 'all' field stems. We have performed this analysis to Figure S3, and referenced it in the limitations paragraph. NEON only samples trees greater than 10cm DBH. In addition, due to the NEON sampling methods this requires some approximations, the details of which are provided in the supplementary material.

We note that, as described in the paper and inline with most airborne remote sensing work, this dataset is not intended to incorporate understory trees. Therefore this analysis is added as a side note to remind potential data users that this is data on canopy trees and so will only reflect all trees in open canopy areas.

b) Under-estimation of species richness by comparing for each site the total number of surveyed tree species in the field (i.e. without thresholds (see specific comment in Table 2).

We have added a modified version of Figure 3 to the supplement that removes the canopy species filter.

Overall, as a user I would like to know how many real trees you are correctly identifying and how many are you missing by using a deep learning model that relies on RGB data.

As described above we appreciate the reviewer's concern about the downstream use of the dataset. Since the paper is about canopy trees, as in almost all image based remote sensing research, evaluating performance based on non-canopy trees is not equivalent. Any measurement between ground-based data and airborne prediction will be difficult to disentangle the differences in perspective, visibility and algorithm performance. It is not clear whether the proportion of underprediction in Figure S3 is due to subcanopy tree abundance, versus the algorithm missing individual trees. The analyses added in response to the reviewer's comments simply reinforce that a dataset on canopy trees should not be used to make inferences about non-canopy trees. In the manuscript we have further reinforced the point that these airborne surveys are not a replacement for field surveys. They are a complement to those surveys that allow the measurement of some key aspects of forests at scales that are not possible using detailed field surveys.

- The introduction contains a large portion of text (lines 71-108) that I feel should belong to the materials and methods rather than the introduction as it describes the data and methods used to derive these data. As a matter of fact, much of the same information is repeated in the methods section. The above points make it rather difficult to clearly understand what the contribution is compared to their previous work. I think that the authors should re-structure the introduction by moving the portion mentioned above to the appropriate section (Materials and Methods) and instead of this provide a more in-depth review of the work done in these past years on single tree detection using aerial imagery or lidar, highlighting the currents gaps and steps that the authors consider important to fill these gaps.

We agree and have thoroughly revised the Introduction and moved a portion of the previous text to the Methods. The new introduction focuses more on the broader themes suggested by the

reviewer, highlighting previous work by the community, as well as previous evaluation of our workflow.

- The methods section is too long and rather intricate and while I agree on the need for complexity behind certain modelling choices, I think that the choice of the authors to train site-specific models is rather odd as it results in a large number of models that are overfitted to each site. While this is unsurprisingly likely to provide better results for that site (overfitted model), it creates confusion for the reader and for the user who will not know which model to apply to any new given area. While this is not a big issue in the case where that the authors only aim at creating the best possible dataset, it is a valid concern in case that the authors will publish the developed methods (which I think is necessary for publication) and trained models.

As noted in our response to Reviewer 2, the focus of this paper is to provide researchers at NEON the best possible data. Our decisions regarding modeling approach emphasize predictive accuracy over transferability. Comparing models based on different underlying data is not yet the focus of our modeling approach. We have made this clearer in text, L211, L486, Figure S2. We are certainly interested in model transferability outside of NEON and are pursuing this in other work. We have also added a figure and text in the supplementary materials L783, Figure S1, to show that initial attempts at RGB models were not successful.

The modeling approach emphasizes predictive accuracy over transferability. This does not mean the models are “overfit”. It means they are tailored to provide the best solution for each individual site. They have been evaluated on held-out data at each site. While transferability is valuable, and we have published a number of papers pursuing this goal in airborne remote sensing (Weinstein et al. 2020, 2021, 2022, Marconi et al. 2021), it is not the goal of this paper and our methods reflect that. To make the justification for our approach clearer, while also clarifying its limitations, we have revised the associated text and highlighted the limitations resulting from the hierarchical approach on L484. We have also added a new supplementary analysis demonstrating the benefits of the approach for improving prediction (Figure S2).

We also note that the paper does not recommend transferring these models to other locations, and while we do release the models, we also release all of the training data used to generate them. This allows others to train a general model if they want to. A general, transferable, species classification model, that can work for even relatively uncommon species, is something we are actively pursuing. However, our current assessment is that doing this will require more data collection, algorithmic development, and mechanistic understanding. We have added the idea that this is an important next step to the Discussion.

- Results: The methods section is a bit long and could benefit from shortening it.

We have reduced the overall manuscript length by more than 1000 words, including tightening the existing language in the Methods section.

- Discussion: This section is also very long and could benefit from shortening it. Here, I think is important that the authors address the quality issues described above (under-detection of trees and under-estimation of species richness) and provide a critical assessment of the limitations of their data and their impact in downstream applications.

We have revised the discussion to be shorter and clearer and added a paragraph highlighting the limitations of the resulting data.

Also, I think that the authors should mention amongst further improvement the fact that one should move away from having many site-overfitted models (as in this study) towards more general models that can predict a broad range of species across biomes. These have the great benefit that they are very versatile in terms of deployment, which becomes key for the model to be used effectively without the users having to select from a long list of models and not knowing which is best suited to their data.

We agree with the goal (see response above) and have added this to L488.

- Methods availability: as mentioned above, I think that for publication the authors should publish the associated code as it is the only proof of the inner working of the methods proposed.

We definitely agree. The code has been open-source and online since we started the project, but we failed to provide a link to it in the paper. We thank the reviewer for catching this oversight. The code is archived on Zenodo (https://zenodo.org/records/7301868) and also available on GitHub as a git repository (https://github.com/weecology/DeepTreeAttention). We have added the appropriate links to the data availability statement on L529.

Changes in response to comments in the PDF document.

A number of the editorial suggestions from the reviewers PDF applied to text that was significantly revised or removed when making the requested changes to the Introduction and Discussion. In those cases we have not provided point by point responses. Point by point responses where appropriated follow below.

I think that these cannot be defined as individual tree maps as what is produced in this dataset is polygons that can have multiple trees or only portion of trees (e.g. one branch). In this sense it is incorrect to refer to these as individual trees. Please revise.

There are several points at which the reviewer objects to our use of the term 'individual', since the tree detection algorithm is imperfect. However, the tree detection model is specifically designed to detect individual trees, which is what determines the language associated with the predictions, not whether or not those predictions are perfect. Our language aligns with a large literature on individual tree detection using imperfect models (https://scholar.google.com/scholar?hl=en&as_sdt=0%2C10&q=individual+tree+detection&btnG=). Therefore it would confuse readers if we deviated from this conventional approach and referred to predictions as something other than what the model is attempting to predict. That said, we again appreciate the reviewer's goal of avoiding confusion by downstream users and have added the word 'predicted' whenever reasonable, including in the title, to highlight that these are not direct measurements of trees, but machine-generated predictions with associated uncertainty and bias.

Instead of the State column, which is not particularly informative, provide a column describing the forest type and climatic region. These would help to better understand in which ecosystems these models perform best."

We have added a site-level description column to Table 2. For many sites, this is quite general as they have multiple forest types and species assemblages.

“please explain better what the rank means”

We **added an additional explanation of this to the Figure 7 legend.**

‘*I think these data are far from the "best possible characterization of canopy tree biodiversity". Please tune down this sentence and in general the discussion.*’

The reviewer has misinterpreted this sentence by reading a portion of it out of context. The complete original sentence was:

“Focusing on optimal end-to-end predictive workflows also highlights the importance of viewing these data as the first step in an ongoing iterative process for generating the best possible characterization of canopy tree biodiversity across increasingly larger areas.”

The sentence does not state the current state of the model is the best characterization. Our aim is to highlight the need for additional data collection (e.g L450) only the first iteration, and that more work needs to be done to produce the best categorizations possible. The route forward is then outlined in the rest of the paragraph.
